# NIR Ratiometric Fluorescent Antibody‐Drug Conjugate for Metastatic Ovarian Cancer Theranostics and Treatment Response Monitoring

**DOI:** 10.1002/advs.202516607

**Published:** 2025-11-28

**Authors:** Cheng Li, Zezhong Yu, Tao Pu, Yanling Wu, Gang Wang, Youhua Xie, Tianlei Ying, Zhenlin Yang, Yibing Shi

**Affiliations:** ^1^ Shanghai Institute of Infectious Disease and Biosecurity Institute for Translational Brain Research, MOE Frontiers Center for Brain Science Obstetrics & Gynecology Hospital of Fudan University School of Basic Medical Sciences Fudan University Shanghai 200032 China; ^2^ Department of Pulmonary Medicine Zhongshan Hospital Fudan University Shanghai 200032 China; ^3^ Shanghai Key Laboratory of Lung Inflammation and Injury Shanghai 200032 China; ^4^ Shanghai Engineering Research Center for Synthetic Immunology Shanghai 200032 China

**Keywords:** fluorescent antibody‐drug conjugate, in situ treatment response monitoring, near infrared bioimaging, ovarian metastases treatment, ratiometric probe

## Abstract

In vivo optical imaging advances antibody‐drug conjugate (ADC) optimization by enabling real‐time monitoring of biological stimulus‐responsive drug behavior. Ratiometric probes, incorporating orthogonal wavelength‐encoded multicolor detection and stimuli‐cleavable linkages, are preferred over single‐color strategies. Here, the synthesis of n501‐CYMMAF (cyanine‐decorated Monomethylauristatin F) is described as a novel ratiometric theranostic agent for dual efficacy in ovarian metastasis treatment and in situ treatment response monitoring. With low‐dose administration (2.5 mg kg^−1^), exceptional target affinity (EC_50(n501‐CYMMAF)_ = 1.17 nm), and favorable biosafety (delivery to tumor‐parts only), n501‐CYMMAF enables experimental verification of treatment‐evading metastatic cell populations through in situ fluorescent tracking, providing crucial insights into tumor recurrence pathways and judgement of treatment terminal point. In general, n501‐CYMMAF represents a prototype for next‐generation smart theranostics, integrating treatment and endpoint assessment to offer a platform for precision metastasis management.

## Graphical abstract

In vivo optical imaging enhances antibody‐drug conjugate (ADC) optimization by enabling real‐time monitoring of stimulus‐responsive drug behavior. We report n501‐CYMMAF, a ratiometric theranostic with strong target affinity and low‐dose efficacy for ovarian metastasis. Its dual therapeutic and imaging functions allow in‐situ tracking of treatment‐evading cells, offering insights into recurrence and treatment endpoints. n501‐CYMMAF exemplifies next‐generation precision metastasis theranostics.

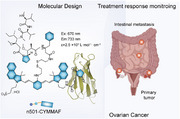

## 1 Introduction

Antibody‐drug conjugates (ADCs) have emerged as a major class of therapeutics in oncology, with currently approved and dozens more in clinical development.^[^ ^1^, ^2^ ^]^ ADCs consist of cytotoxic agents covalently linked to antibodies for targeted drug delivery, enabling 100‐1000‐fold higher payload accumulation in tumors compared with conventional chemotherapies.^[^ ^3^ ^]^ Many FDA‐approved ADCs contain cleavable linkers that respond to intracellular triggers (e.g., lysosomal acidic pH, enzymatic targets like cathepsins) for drug release.^[^ ^4^ ^]^ However, IgG‐based ADCs often face limitations in solid tumors due to poor tissue penetration and Fc‐mediated off‐target effects.^[^ ^5^ ^]^ To address these issues, next‐generation ADCs incorporate antibody fragments (e.g., Fab, scFv, VHH, etc), humanized single‐domain antibodies, dual‐payload systems combining microtubule disruptors (e.g., MMAE, MMAF, etc) and DNA‐damaging agents (e.g., SN‐38, DXd, etc).^[^ ^6^, ^7^ ^]^ Mirvetuximab soravtansine, targeting folate receptor α, exemplifies this progress in platinum‐resistant ovarian cancer by delivering localized cytotoxicity with reduced systemic toxicity. Ultimately, ADC efficacy depends on the interplay among antibody, linker, payload, and tumor microenvironment, underscoring the value of real‐time activity monitoring for clinical optimization.

Despite recent advances in cancer therapy, metastasis, resulting from malignant cell dissemination from primary tumors, still accounts for over 90% of cancer‐related deaths.^[^ ^8^ ^]^ While contemporary therapies successfully eradicate localized malignancies, five‐year survival rates remain dismal for metastatic disease due to systemic progression.^[^ ^9^ ^]^ Advanced ovarian cancer exhibits widespread peritoneal dissemination, high recurrence rates, and poor long‐term survival despite initial response to debulking surgery and platinum‐based chemotherapy.^[^ ^10^ ^]^ Most patients relapse within two years, and the development of chemoresistance limits subsequent therapeutic options. Consequently, novel targeted therapies and more sensitive diagnostic tools are essential for improving clinical outcomes. Concurrently, advances in high‐sensitivity molecular imaging enable the detection of subclinical or microscopic metastatic lesions, a major cause of early recurrence undetectable by conventional imaging.^[^ ^11^ ^]^ Techniques like antibody‐based positron emission tomography (immuno‐PET) or near‐infrared fluorescence imaging facilitate early diagnosis, surgical guidance, drug delivery monitoring, and therapeutic response assessment.^[^ ^12^ ^]^

Biological stimulus‐responsive in vivo optical imaging offers a robust platform for refining ADC architectures by dynamically mapping drug payload release and target engagement.^[^ ^13^ ^]^ With original and discrete wavelengths combined for multicolor imaging, ratiometric probes with activatable linkages and labile groups are more welcomed than single‐color strategies and radiolabeling methods.^[^ ^14^, ^15^ ^]^ These strategies have the potential to real‐time assess local chemical environments encountered by ADCs, with abundant information about both their design and use.^[^ ^16^ ^]^ Further probe discovery efforts are needed to facilitate such studies.

**Scheme 1:**
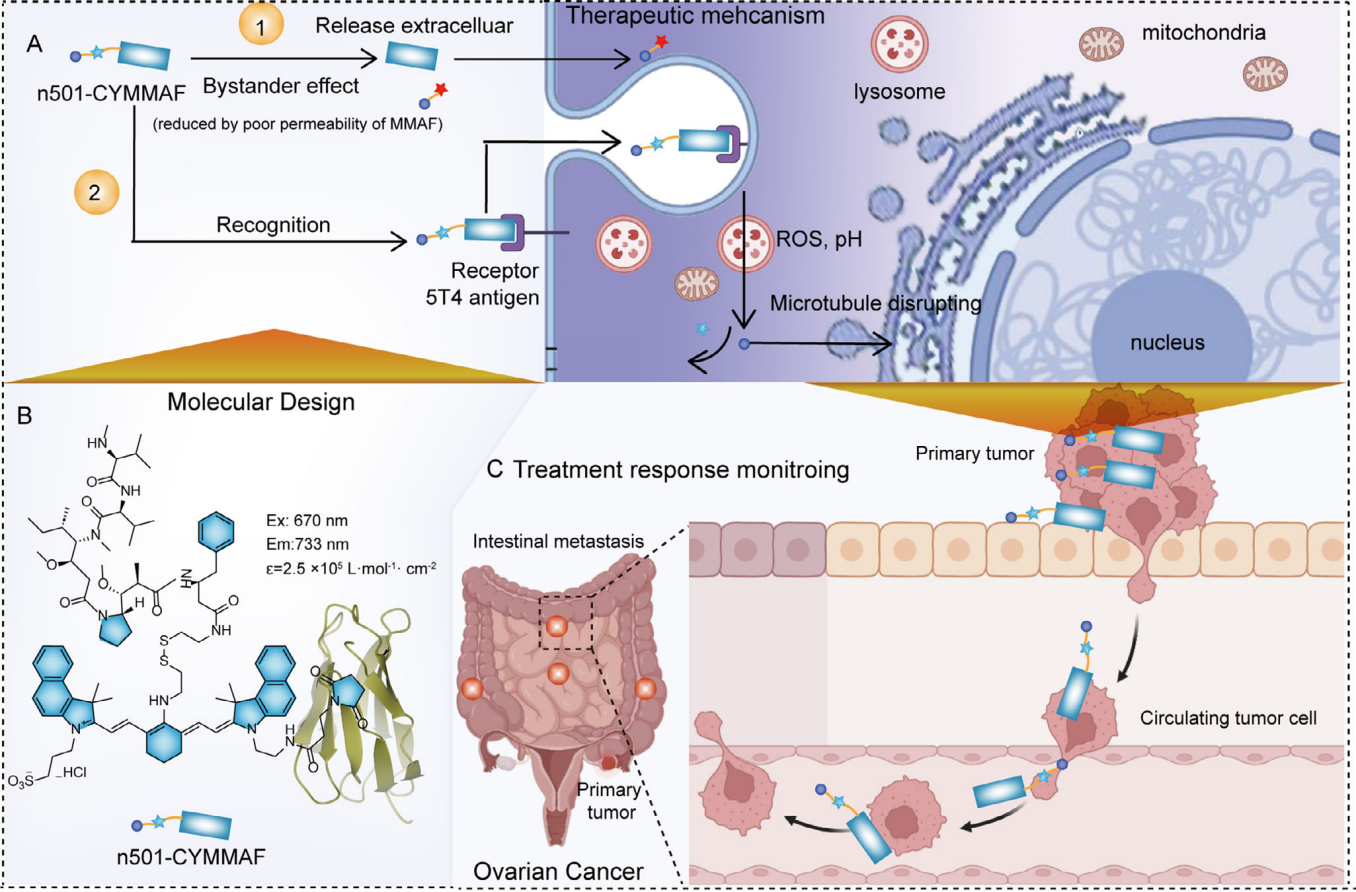
A) Illustration of the ratiometric ADC cellular functioning process. B) Structure design of n501‐CYMMAF. C) Illustration of metastatic ovarian cancer treatment and evading circulation cells tracking.

Here, we report a ratiometric theranostic ADC (n501‐CYMMAF) constructed using a clickable disulfide‐modified cyanine linker bridging a fully human single‐domain antibody (n501) and the cytotoxin MMAF (**Scheme** **1**). Upon conjugation, excitation shifts from 670 to 528 nm, enabling visualization of microenvironment‐triggered drug release and tracking of residual tumor cells. n501‐CYMMAF combines potent antimetastatic efficacy with real‐time mechanistic monitoring, achieving high target affinity (EC_5_₀ = 1.17 nm) and effective tumor suppression at low doses (2.5 mg kg^−1^). Its ratiometric fluorescence further allowed direct identification of therapy‐evading metastatic cells through in situ immunofluorescence, offering mechanistic insights into recurrence. Collectively, n501‐CYMMAF represents a prototype for next‐generation smart theranostics, integrating targeted treatment and dynamic imaging for precision management of metastatic ovarian cancer.

## 2 Results and Discussion

### 2.1 5T4 Antigen is Overexpressed in Ovarian Tumor Cells

**Figure 1:**
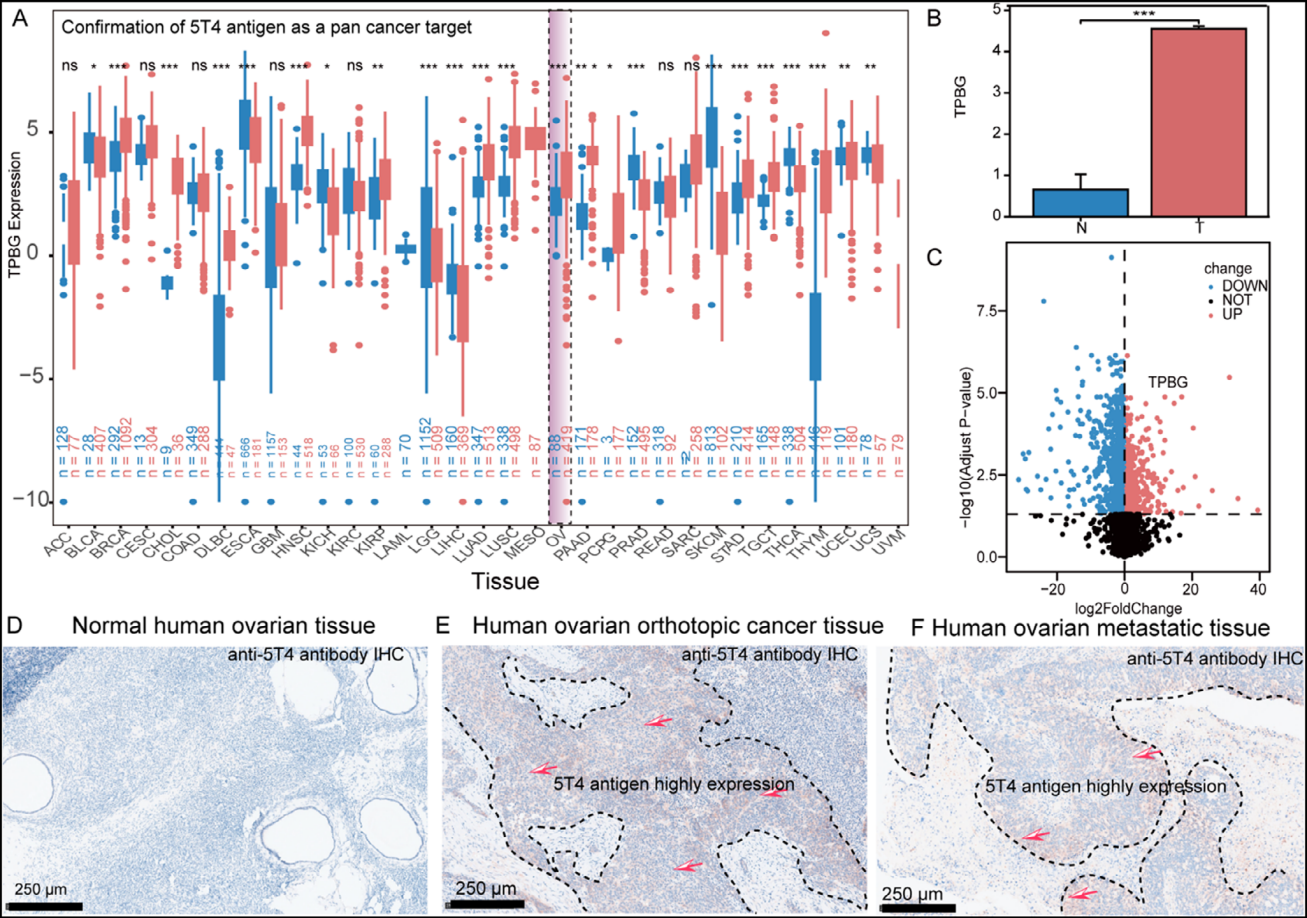
5T4 is a pan‐cancer marker. A) Confirmation of 5T4 antigen as a pan‐cancer target. B) Group comparison chart of TPBG in ovarian cancer cells from TCGA and GEO databases. C) and the corresponding volcano plot. D–F) IHC analysis of tissues from normal human ovary (D), human ovarian orthotopic tumor (E), and human ovarian metastasis tumor (F). Staining of 5T4 is indicated by red arrays.

Pan‐cancer studies suggest that targeting shared molecular traits across cancer types could enable universal therapeutic strategies. The oncofetal antigen 5T4, encoded by TPBG gene, is highly and selectively expressed in tumor cells, making it an attractive ADC target.^[^ ^17^ ^]^ To confirm its overexpression in diverse tumor types, we analyzed TPBG gene levels using the online Gene Expression Profiling Interactive Analysis (GEPIA) platform (**Figure** **1**A, http://gepia.cancer‐pku.cn/). Among the 33 kinds of tumor types analyzed, TPBG gene was significantly up regulated in 17 kinds and down in 3. In particular, TPBG gene showed a significantly higher level in ovarian cancer tissues than in normal ovary (Figure 1B,C) based on TCGA and GEO dataset (GSE14407). To further validate this conclusion, Immunohistochemical staining further confirmed strong 5T4 expression in ovarian orthotopic and metastatic tumor tissues, but not in normal ovary (Figure 1D,E). In general, 5T4 antigen represents a promising target for ovarian cancer treatment and metastatic prognosis evaluation.

### 2.2 Synthesis of CYMMAF

**Figure 2:**
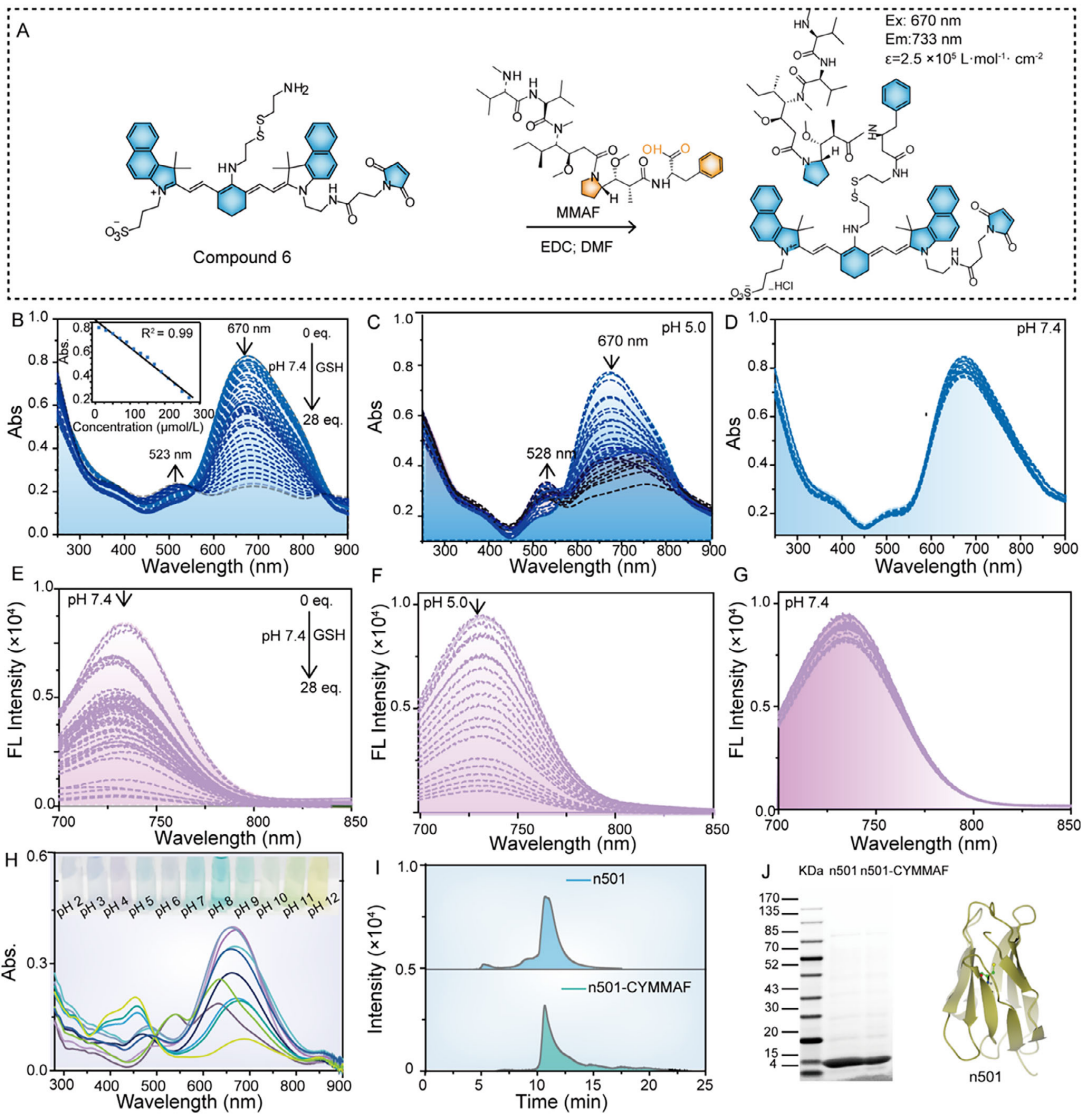
Optical properties of n501‐CYMMAF. (A) Key synthetic procedure from Compound 10 to CYMMAF. (B) UV–Vis and (E) fluorescent spectra of CYMMAF (10 µm) upon addition of cysteine (280 µm in total) within the solution of EtOH/PBS (v/v;1/4); (Inset B) The calibration lines fitted based on the concentrations of cysteine with the absorbance at 679 nm of CYMMAF. (C) UV–Vis and (F) fluorescent spectra of CYMMAF (10 µm) at different time (1.6 h in total) within the solution of DMSO/pH 5 buffer (v/v;1/4); (D) UV–Vis and (G) fluorescent spectra of CYMMAF (10 µm) within the solution of EtOH/PBS (v/v;1/4), (H) UV–Vis spectra of Compound 10 in different buffer pH buffer (2‐12) conditions. (I) HPLC measurement of n501 and n501‐CYMMAF. (J) SDS‐Page and structure diagram of n501.

Advancements in linker chemistry have been pivotal to ADC success, balancing plasma stability with efficient tumor‐specific drug release. Cleavable linkers exploit tumor‐associated triggers such as acidic pH (hydrazones), reducing environments (disulfides), or enzymatic activity (peptide linkers), as exemplified by brentuximab vedotin and trastuzumab deruxtecan.^[^ ^6^ ^]^ For this construct, here we summarized the clinically approved ADC drugs (Figure S1, Supporting Information) and chose monomethyl auristatin F (MMAF), a potent tubulin polymerization inhibitor with a modifiable carboxyl group, for further fluorescent ADC design.^[^ ^18^, ^19^, ^20^, ^21^, ^22^, ^23^, ^24^, ^25^, ^26^, ^27^, ^28^, ^29^, ^30^, ^31^, ^32^, ^33^, ^34^ ^]^ Compared with MMAE, MMAF exhibits lower systemic toxicity due to greater hydrophilicity and reduced aggregation. However, its pharmacokinetics and metabolic behavior remain insufficiently characterized. To address this, we designed CYMMAF employed a disulfide bridge between MMAF and cyanine dye CY7.5 (Figures S2–S5, Supporting Information), enabling dual pH/GSH‐responsive drug release. The synthesis faced key purification challenges with Compound 10 (**Figure** **2**A; Figures S6 and S7, Supporting Information) and CYMMAF (Figures S8 and S9, Supporting Information). Cystamine dihydrochloride altered the intermolecular charge transfer of heptamethine cyanine dye Compound 9, inducing a diagnostically useful color shift from green to blue that facilitated TLC‐based separation. CYMMAF was subsequently synthesized via conjugation of Compound 10 with MMAF and purified by gravity chromatography under UV–vis monitoring (λ_ex_ = 670 nm), yielding a gray‐blue product confirmed by comprehensive ^1^H and ^13^C NMR analysis.

### 2.3 Optical Characterization of CYMMAF

To evaluate physiological relevance, we performed detailed GSH titration studies under tumor‐mimetic conditions (pH 5) and plasma‐equivalent pH 7.4 (Figure 2B,C). Both conditions induced structural degradation of CYMMAF, evidenced by 670 nm absorbance reduction and concomitant 523 nm peak emergence. Notably, CYMMAF demonstrated plasma stability at pH 7.4 without GSH, showing maintained absorption at 670 nm – a critical feature for tumor‐specific cytotoxicity (Figure 2D). Parallel fluorescence monitoring (630‐nm excitation, 680‐nm longpass filter) confirmed the same trends, such as GSH exposure at both pH levels caused 730 nm emission attenuation (Figure 2E,F), as well as physiological pH alone preserved spectral integrity (Figure 2G). This dual pH and redox responsiveness aligns with the acidic, GSH‐enriched tumor microenvironment, supporting the potential of CYMMAF as a ratiometric probe for ADC mechanism monitoring. The ratiometric probe capability of CYMMAF was preliminarily assessed through UV–vis spectral analysis across pH 2–12 buffers (Figure 2H), revealing characteristic blueshifts from 670 to 537 nm (acidic), 482 nm, or 458 nm (basic).

### 2.4 Synthesis and Characterization of n501‐CYMMAF

**Figure 3:**
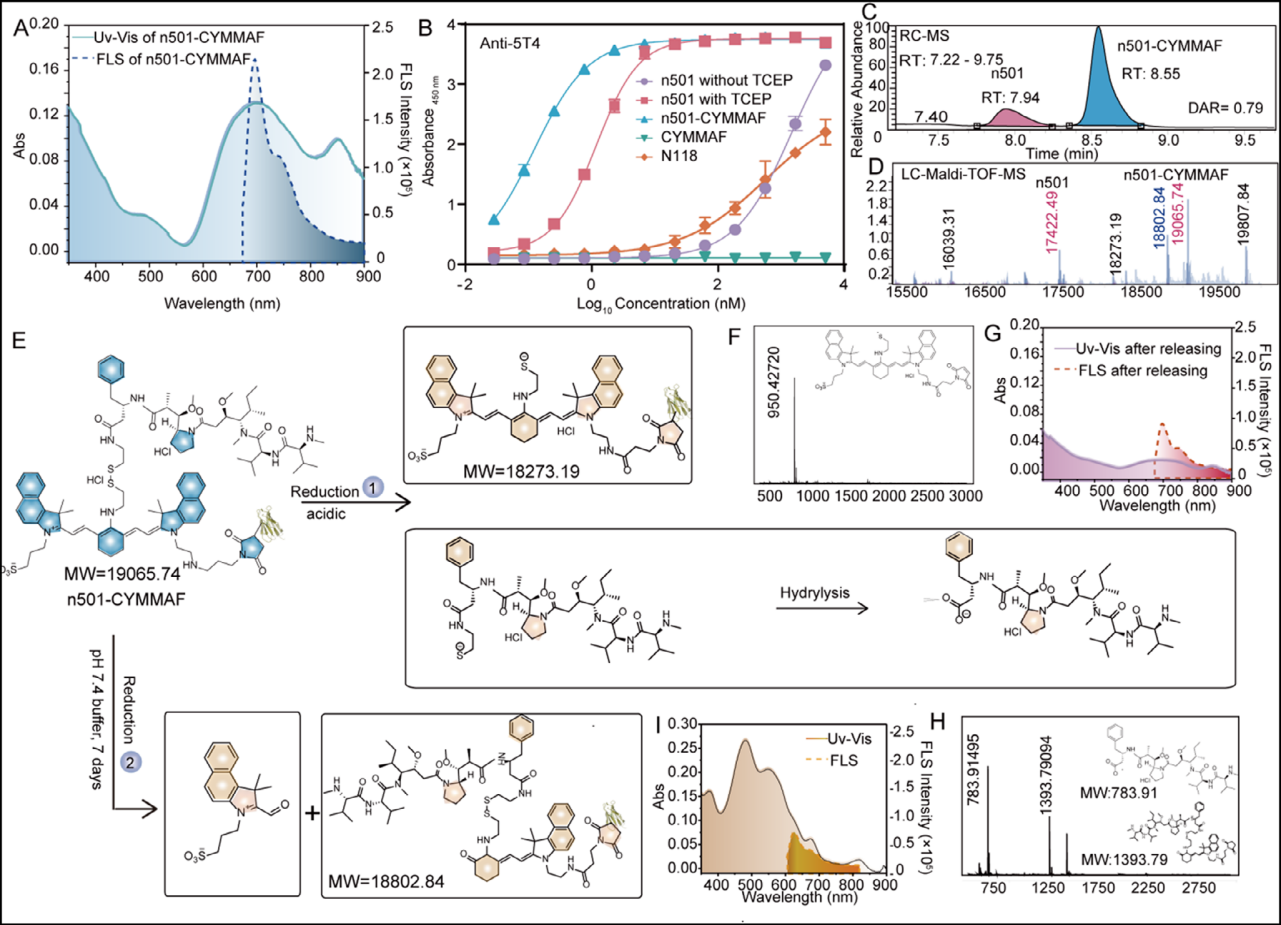
Characterization of n501‐CYMMAF and mechanism illustration. A) UV–vis and fluorescent spectra of n501‐CYMMAF. B) Binding affinity tests between 5T4 and n501‐CYMMAF, n501 with and without TCEP, CYMMAF, and an irrelevant sdAb n118 based on ELISA (n = 3, and the error bars represent the standard deviation of the two absorbance values measured at 405 nm). C) Drug‐to‐antibody (DAR) ratio calculation based on MS and D) LC‐MS‐TOF monitoring of n501‐CYMMAF deterioration. E) Mechanism illustration of n501‐CYMMAF releasing MMAF in vitro based on the F) LC‐MS‐TOF of reduction byproduct CY and G) UV–vis and fluorescent spectra of byproduct in one pathway. H) LC‐MS‐TOF monitoring of n501‐CYMMAF lingering moiety, and I) the UV–vis and fluorescent spectra helped in demonstration of the other way.

We previously constructed a fully human single domain antibody library from healthy donor peripheral blood B cells and identified a 5T4‐targeting single domain antibody (sdAb), designated as n501.^[^ ^35^ ^]^ Ser85 outside of the antigen‐binding region was chosen to generate a free cysteine for the conjugation of CYMMAF. During manufacturing, TCEP prevented cysteine crosslinking while preserving site‐specific conjugation capacity. These features collectively position n501 as a versatile platform for precision therapeutics and diagnostics, as previously demonstrated in in vivo ovarian metastasis imaging. This nanobody was specifically engineered to target 5T4 and contains a single cysteine residue located outside its functional region, enabling site‐specific drug conjugation (patent application No. 201611040981.8). To improve the aqueous solubility of MMAF, we optimized the synthesis of n501‐CYMMAF. Sequential gradient centrifugation (4000, 10 000, and 12 000 rpm, 5 min per step) effectively removed unreacted dyes and degraded sdAbs. Subsequent purification steps included dialysis for buffer exchange and desalination chromatography to eliminate low‐molecular‐weight impurities. Final product concentration was quantified using HPLC and SDS‐PAGE (Figure 2I,J), confirming high purity of the conjugate. UV–vis and fluorescence spectra confirmed structural integrity (**Figure** **3**A), while ELISA demonstrated preserved 5T4‐binding affinity after conjugation (Figure 3B). RP‐LC‐MS and LC‐MS analyses determined a drug‐to‐antibody ratio (DAR) of 0.79 and a molecular weight of 19065.70 Da (Figure 3C,D). While conventional thiol‐maleimide conjugation often produces heterogeneous mixtures with varying drug‐to‐antibody ratios (DARs, typically 0–8), leading to inconsistent pharmacological properties and reduced stability, the n501 design restricts the DAR to 0–1. Consequently, the observed DAR of 0.8 was relatively high for this vehicle, and the one‐to‐one conjugation strategy ensured excellent batch‐to‐batch reproducibility throughout all syntheses. Two distinct payload release pathways were identified (Figure 3E), and validated by UV–vis and fluorescence spectra (Figure 3G,I) and LC‐MS analysis (Figure 3F,H; Figure S10, Supporting Information). Under acidic conditions, the disulfide bond was cleaved, yielding two primary products: a cyanine‐cystamine conjugate (950 Da) and free MMAF (783 Da) (Figure 3F,H). The corresponding degradation was monitored by UV–vis absorption at 670 nm and fluorescence emission at 730 nm. In contrast, under physiological conditions (pH 7.4), oxidative cleavage of the C═C bond generated a keto derivative and fragmentation of the Cy7.5 moiety, yielding two products characterized by absorption at 482 nm and emission at 630 nm. In addition, to calibrate the fluorescence ratio with cleavage efficiency, anti‐MMAF mAb was used to quantify MMAF content in n501‐CYMMAF under different conditions (Figure S11, Supporting Information), confirming its ratiometric property. Worth mentioning, the coupling of the antibody to CYMMAF induced a redshift in absorbance between 800 and 900 nm (Figure 3A,I), attributed to the twisted intramolecular charge transfer (TICT) process.

### 2.5 Cytotoxicity, Cellular Bioaffinity, and Half‐Life of n501‐CYMMAF

**Figure 4:**
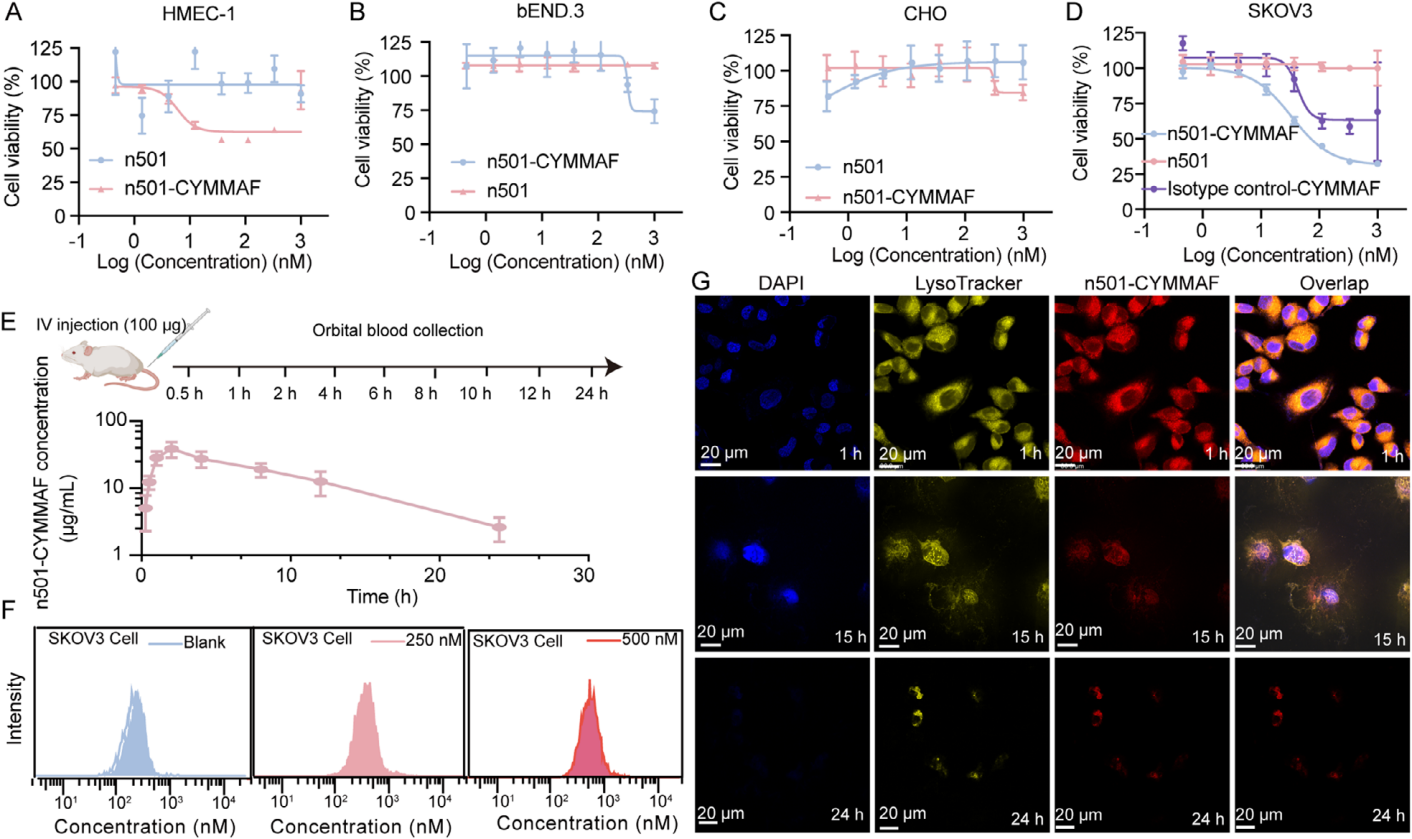
Ex vivo cytotoxicity and cellular bioaffinity of n501‐MMAF. A–D) Cell cytotoxicity assays performed on (A) HMEC‐1 cells, (B) bEND.3 cells, (C) CHO cells, and (D) SKOV3 cells, treated with the indicated antibody drugs (n = 3, and the error bars represent the standard deviation of the two absorbance values measured at 450 nm).(E) The Pharmacokinetics based on the anti‐MMAF mAb for quantification. F) Flow cytometer‐based cellular bioaffinity of n501‐CYMMAF (0, 250, and 500 nm) to SKOV3 cells. G) Confocal bioimaging of n501‐CYMMAF (400 nm) cultured with SKOV3 cells for 1, 15, and 24 h, with lysoTracker at 590 nm and n501‐CYMMAF at 650 nm for emission collection.

Ex vivo cytotoxicity was assessed by cytotoxicity assays and confocal bioimaging. N501‐CYMMAF showed strong cytotoxicity toward 5T4‐positive SKOV3 cells, weaker effects on HMEC‐1 cells, and minimal to no toxicity on CHO and bEND.3 cells, suggesting that cell line origin influences sensitivity. The conjugation site between Cy7.5 and MMAF was the C‐terminal carboxyl group of MMAF, slightly enhancing membrane permeability by reducing electronegativity. As a control, an irrelevant sdAb‐CYMMAF targeting type III collagen showed only weak cytotoxicity toward SKOV3 cells at high concentrations (**Figure** **4**D). For pharmacokinetic evaluation, blood samples collected over 0–24 h were analyzed using an anti‐MMAF mAb with anti‐Flag HRP detection (Figure 4E) and fluorescent intensity (Figure S12, Supporting Information). The intact conjugate reached peak plasma concentration ≈2 h post‐injection, with a half‐life of ≈5.4 h for n501‐CYMMAF. Flow cytometry further confirmed the binding of n501‐CYMMAF to SKOV3 cells at 250 and 500 nm (Figure 4F).

As demonstrated in Figure 4D, SKOV3 cells (selected for their high 5T4‐antigen expression level) were treated with lysoTracer (excitation at 590 nm) to enable precise lysosomal colocalization tracking. The therapeutic mechanism involves lysosomal proteolytic cleavage of the antibody‐payload covalent linkage, facilitating payload release from lysosomal compartments and subsequent engagement with intracellular molecular targets. Time‐course analysis revealed rapid lysosomal accumulation of n501‐CYMMAF within 1‐h post‐treatment, followed by efficient cleavage that mediated progressive cellular damage over 24 h. Through simultaneous monitoring of n501‐CYMMAF/lysosomal colocalization dynamics and subsequent cellular deterioration following CYMMAF release, we successfully demonstrated the dual functionality of this conjugate: direct cytotoxic effects coupled with lysosomal degeneration capacity. This parallel assessment provides mechanistic insights into both the therapeutic efficacy and lysosomotropic activity of this drug. For biosafety evaluation, H&E staining and blood tests were performed (Figures S13 and S14, Supporting Information). The ADC‐treated group exhibited a slight increase in neutrophil count and a mild decrease in ALBP levels, while all other parameters remained comparable to those of the control group.

### 2.6 In Vivo Diagnosis and Treatment in Ovarian Cancer Peritoneal Metastasis Model

**Figure 5:**
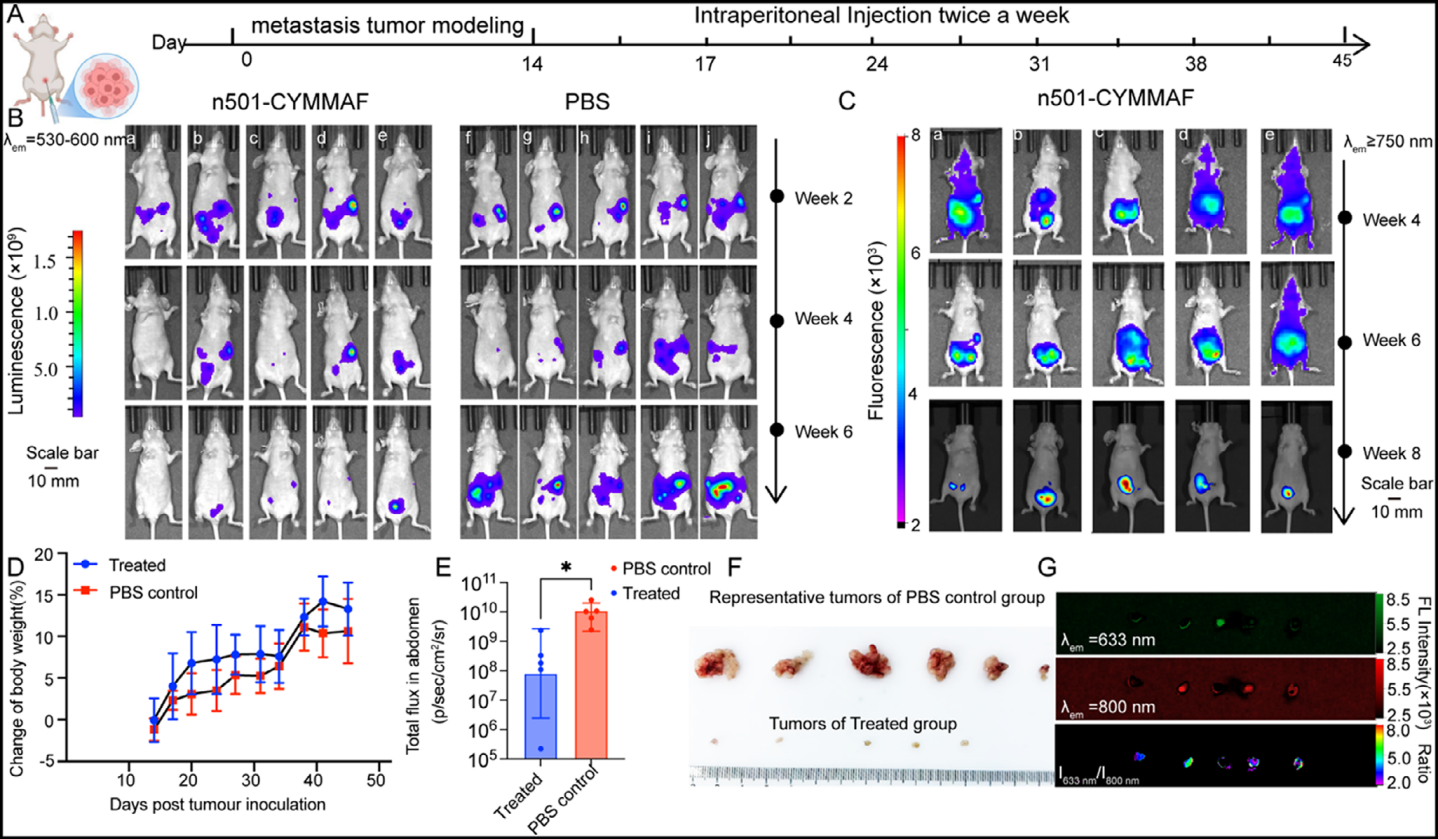
In vivo therapeutic efficacy of n501‐CYMMAF in an ovarian cancer peritoneal metastasis model. A) Treatment schedule for n501‐CYMMAF (5 mg Kg^−1^) and PBS (100 µL mouse^−1^). B) Luminescent bioimaging of mice intraperitoneally injected with SKOV3 cells and treated with n501‐CYMMAF or PBS. C) Fluorescent bioimaging of n501‐CYMMAF‐treated mice. D) Body weight changes (n = 5; mean ± SD). E) Quantification of abdominal total flux analyzed by one‐way ANOVA with Bonferroni correction. F) Representative tumors from PBS and n501‐CYMMAF‐treated mice. G) Fluorescent imaging of resected tumors at 633 nm, 800 nm, and merged channels. The exposure times were 5 s for Figure 5B,C, 20 s for Figure 5G.

**Figure 6:**
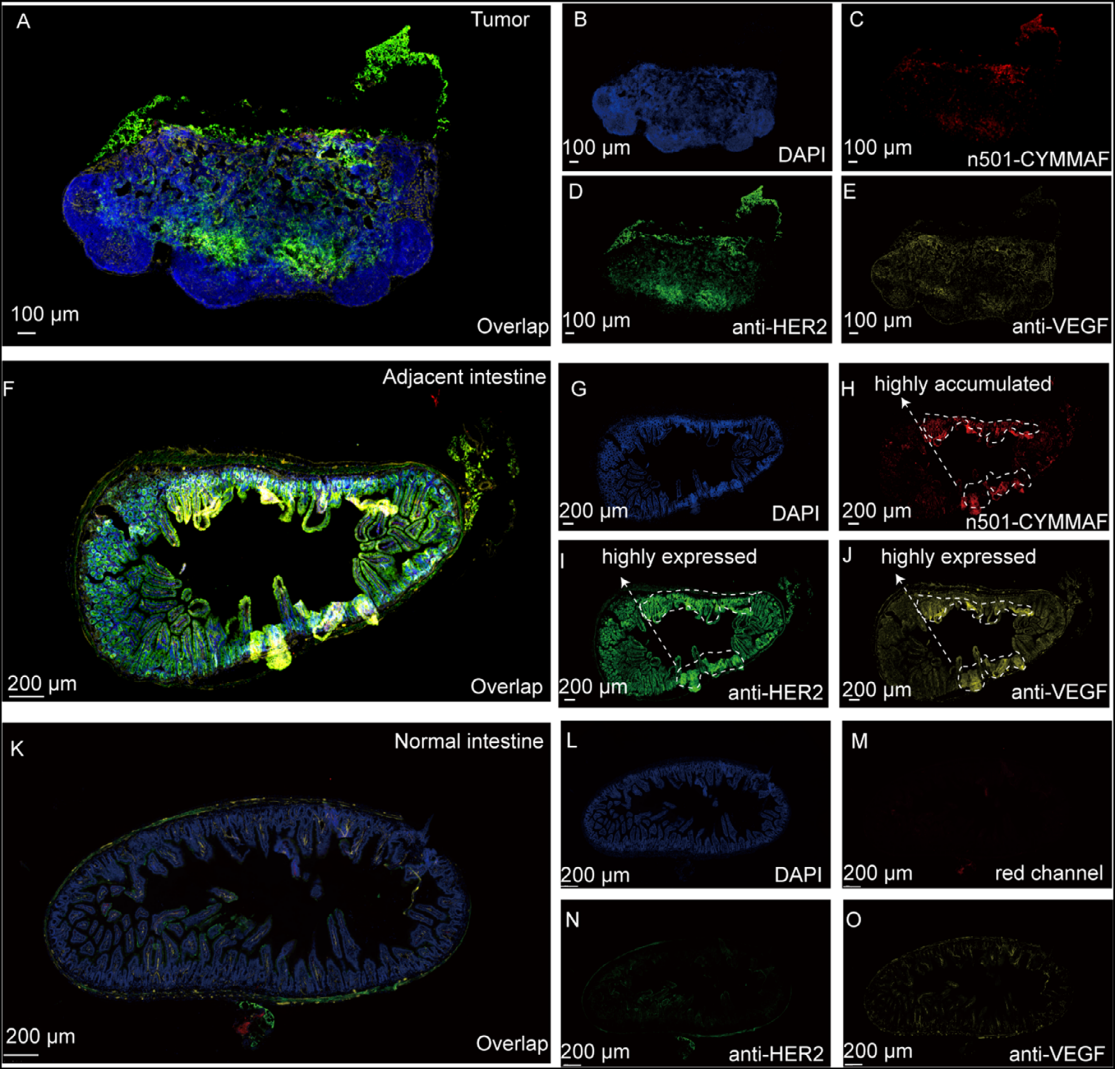
In situ analysis of metastasis lesion. A) Immunofluorescent staining image of tumor from mouse b, B) the DAPI‐channel based image, C) remaining n501‐CYMMAF in the tumor‐based image, D) anti‐HER2‐channel based image, E) anti‐VEGF‐channel based image. F) Immunofluorescent staining image of intestine adjacent to tumor lesion, G) the DAPI‐channel based image, H) remaining n501‐CYMMAF in the tumor‐based image, I) anti‐HER2‐channel based image, J) anti‐VEGF‐channel based image. K) Immunofluorescent staining image of intestine adjacent to tumor lesion, L) the DAPI‐channel based image, M) remaining n501‐CYMMAF in the tumor‐based image, N) anti‐HER2‐channel based image, O) anti‐VEGF‐channel based image.

As a theranostic agent integrating diagnostic and therapeutic functions, n501‐CYMMAF was evaluated in a metastatic ovarian cancer model using nude mice bearing SKOV3/luc xenografts. The intrinsic fluorescence from this compound enabled real‐time in situ tumor monitoring, while luciferin‐based bioluminescence imaging served as an accuracy control. Mice received intraperitoneal injections of either n501‐CYMMAF (5 mg Kg^−1^) or PBS (100 µL) two times per week for 45 days (**Figure** **5**A). Imaging analysis revealed that fluorescence signals encompassed bioluminescence‐defined tumor regions while covering a broader anatomical area (Figure 5B,C). Fluorescence imaging with n501‐CYMMAF consistently detected tumor lesions for two weeks longer than luciferase‐based bioluminescence imaging (e.g., fluorescence patterns at week 8 spatially matched bioluminescence signals at week 6), indicating broader lesion detection capability (this part would be thoroughly elucidated in **Figure** **6**). Complete signal regression in select mice (marked as a and c) prompted terminal imaging and necropsy. Body weight monitoring demonstrated gradual increases in both treatment and control groups (Figure 5D), potentially attributable to immunotherapy‐induced ascites or metastatic progression. Quantitative analysis of bioluminescence signals under standardized intensity scales revealed significantly higher radiance in PBS controls compared to the n501‐CYMMAF cohort (*p* < 0.05). Postmortem examination confirmed therapeutic efficacy, with PBS‐treated mice exhibiting extensive tumor burden versus rice‐grain‐sized lesions in the treatment group (Figure 5F; Figure S15, Supporting Information). Fluorescence imaging used the 633‐nm channel to monitor drug release and the 780‐nm channel to locate tumor sites (Figure S15, Supporting Information). Excised tumors exhibited dual emissions at 633 and 780 nm, with superior specificity at 780 nm (Figure 5G). Collectively, these findings demonstrate that n501‐CYMMAF functions as a potent theranostic ADC for metastatic ovarian cancer management.

### 2.7 In Situ Analysis of Metastasis Mechanism

For in situ evaluation of mechanisms related to metastasis, treatment was halted before the complete disappearance of tumor bioluminescence. Mice treated with n501‐CYMMAF for 45 days underwent final bioluminescence imaging to confirm metastatic status (Figures S16 and S17, Supporting Information), after which tumors and adjacent tissues were collected for immunofluorescence staining (Figure 6A–F). The residual 5T4‐expressing lesions were visualized via n501‐CYMMAF fluorescence. Immunostaining for HER2 (Figure 6D,I,N) and VEGF (Figure 6E,J,O) further identified residual metastatic cells. Regions with strong n501‐CYMMAF accumulation (Figure 6C) corresponded to elevated VEGF and HER2 expression, indicating synchronized localization among these markers. H&E analysis was applied for confirmation of the tumor (Figure S18, Supporting Information).

To validate the in situ mechanism, we collected the intestinal regions invaded by metastatic tumors previously identified by bioluminescence/fluorescence during the treatment (Figure 6F). Using HER2 to track SKOV3/Luc cells, VEGF to mark proliferating vasculature, and n501‐CYMMAF to monitor therapeutic drugs, the evading ovarian cancer cells could be monitored as a reference indicator for the termination of treatment. Despite bioluminescence disappearance at the final timepoint (Figure S16, Supporting Information), anti‐HER2 and anti‐VEGF fluorescence detected persistent SKOV3 cells. Synchrony persisted between n501‐CYMMAF accumulation and VEGF/HER2 expression, with high‐accumulation regions corresponding to elevated VEGF/HER2 areas. This indicates residual SKOV3/Luc cells and vasculature in the lesion, demonstrating n501‐CYMMAF's ability to target bioluminescence‐undetectable dormant tumor cells. For rigor, normal intestinal tissue from healthy mice (Figure 6K) showed negative signals across all channels versus experimental tissue (Figure 6A,F), confirming HER2/VEGF overexpression evidences residual tumor cells.

This study provides new insights into metastatic ovarian cancer biology. Conventional therapies (surgery, chemotherapy, radiotherapy, immunotherapy) primarily eliminate visible tumors but fail to eradicate circulating tumor cells or minimal residual disease (MRD) in occult or distant sites.^[^ ^18^ ^]^ Our findings highlight this limitation: despite near‐complete primary tumor clearance, IHC revealed persistent disseminated cells, implying latent risk of metastatic relapse. By integrating fluorescence‐based lesion detection with targeted cytotoxicity, n501‐CYMMAF offers a promising theranostic approach for early detection and eradication of residual metastatic disease.

## 3 Conclusion

Despite therapeutic advances in cancer treatment, metastasis remains the principal cause of cancer death. We developed a fully human single‐domain based fluorescent drug conjugate n501‐CYMMAF as a ratiometric theranostic agent for ovarian metastasis therapy and in situ mechanism exploration. With good bioaffinity (EC_50(n501‐CYMMAF)_ = 1.17 nmol L^−1^), ratiometric bioimaging at 633 to 730 nm, n501‐CYMMAF presented excellent curative effect and uncovered the prognosis state of metastasis. By combining dual‐capacity fluorescence imaging (for real‐time tracking of migratory tumor cells and residual microlesions) with tumor‐specific cytotoxicity, this agent enabled simultaneous detection and eradication of occult metastatic foci. Such multifunctionality could revolutionize therapeutic regimens by facilitating early intervention in subclinical metastasis, thereby addressing a critical unmet need in oncology.

## 4 Experimental Section

### Ethics Statement

Human tissue samples were collected with approval from the Clinical Research Ethics Committee of Obstetrics & Gynecology Hospital of Fudan University (Approval Number: 2024–193). All animal procedures were approved by the Fudan University Institutional Animal Care and Use Committee (IACUC) and conducted in accordance with the AVMA Guidelines for the Euthanasia of Animals. At the study endpoint, mice were deeply anesthetized with isoflurane (5% for 2 min). Upon loss of the pedal reflex, euthanasia was completed by discontinuing the oxygen supply, and death was confirmed by the absence of respiration and heartbeat.

### Materials and Instruments

All the reagents and consumables were commercially available. 1,1,2‐trimethyl‐1H‐benz [e] indole, cyclohexanone, N, N ‘‐dimethylformamide, 1,3‐propanesultone, phosphorus oxychloride, acetic anhydride, potassium acetate, sodium bicarbonate, and 2‐bromoethylamine hydrobromide were purchased from J&K Chemicals. Ditertbutyl dicarbonate, N, N′‐diisopropylethylamine, trifluoroacetic acid, N, N‐diisopropylethylamine, 3‐maleimidopropionic acid, O‐(N‐succinimidyl)‐1,1,3,3‐tetramethyl uranium, and tetrafluoroborate were purchased from Bidepharm. Chemical solvents, including toluene, dichloromethane, diethyl ether, chloroform, DMSO, and methanol were purchased from Titan. The single‐domain antibody expressing apparatus was supplied by Thermo Fisher Scientific. ^1^H NMR and ^13^C NMR spectra were recorded on a Bruker AV‐400 spectrometer with chemical shifts reported in ppm (in MeOD or DMSO‐d_6_; tetramethyl silane as the internal standard). Ultra high‐performance liquid chromatograph (Waters, I‐Class BioSystem) was recorded for molecular weight measurement. UV−vis−NIR absorption spectra were recorded using a Shimadzu 3000 spectrophotometer. Emission spectra were recorded using an Edinburgh FS5 luminescence spectrometer. All the cell lines (SKOV3/Luc and bEND.3) were purchased from Gempharmtech Company and have been cultured in Ying's Lab for many years without contamination. All the nude mice for animal experiments were purchased from Gempharmtech. And animal procedures were on the basis of the guidelines from the Institutional Animal Care and Use Committee of Fudan Shanghai Basic Medical College. The related NIR bioimaging was conducted on an IVIS Lumina III apparatus.

### Synthesis of Compound 2

A mixture of N, N‐dimethylformamide (20 mL) and CH_2_Cl_2_ (20 mL) under ice‐cooling was treated dropwise with phosphorus oxychloride (37 mL, 39.7 mol) and CH_2_Cl_2_ (35 mL). Cyclohexanone (10 g, 100 mmol) was rapidly injected into the reaction mixture, followed by reflux at 80 °C for 2 h. The cooled mixture was quenched by pouring into 200 mL of ice water (0 °C), yielding compound 1 as a yellow solid after overnight storage at 4 °C. Separately, a pre‐cooled (0 °C) solution of aniline/ethanol (1:1 v/v) was treated with compound 1 (1:1 molar ratio) and stirred for 1 h. The mixture was acidified with HCl at 0 °C, reacted for 1 h, and stored at 4 °C overnight. The resulting red solid (compound 2) was collected by filtration.

### Synthesis of Compound 5

Compound 3 (2.09 g, 10 mmol) was dissolved in toluene (5 mL) and added dropwise into a round‐bottle containing 1,3‐propanesultone (2 mL, 21.6 mmol) for further 2‐h heating at 110 °C, obtaining compound 4. Compound 2 and potassium acetate were dissolved in acetic anhydride, added in batches to a bottle containing compound 4 and stirred at 80 °C for another 1 h, to obtain compound 5.

### Synthesis of Compound 7

Compound 3 (6.27 g, 30 mmol) and 2‐bromoethylamine hydrobromide (2.04 g, 10 mmol) were ground and mixed, then heated at 140 °C for 2 h. The resulting gray solid (Compound 5) was obtained through crystallization with chloroform. Compound 5 (2.53 g, 10 mmol) and di‐tert‐butyl dicarbonate (2.18 g, 10 mmol) were dissolved in chloroform with N,N‐diisopropylethylamine (0.1 mL, 2.9 mmol) and refluxed at 65 °C. After extraction with diethyl ether (3 × 30 mL), Compound 6 was isolated. Subsequently, compound 4 (0.576 g, 1 mmol), compound 6 (0.353 g, 1 mmol), and potassium acetate (0.196 g, 2 mmol) were stirred in acetic anhydride (10 mL) at 70 °C for 1 h, yielding a green solution. Purification by column chromatography using CH_2_Cl_2_/MeOH (1:10 v/v) afforded the final product. Structural characterization was performed by ^1^H and ^13^C NMR spectroscopy: ^1^H NMR (400 MHz, CDCl_3_) δ = 8.45 (dd, J = 46.0, 14.0 Hz, 3H), 8.11 (t, J = 8.3 Hz, 2H), 7.93 (dd, J = 19.1, 8.2 Hz, 4H), 7.64‐7.55 (m, 3H), 7.51‐7.40 (m, 3H), 6.72 (d, J = 14.5 Hz, 1H), 6.21 (s, 1H), 5.86 (s, 1H), 4.68‐4.58 (m, 2H), 4.36‐4.26 (m, 2H), 3.37 (s, 3H), 3.12‐3.06 (m, 2H), 2.92‐2.85 (m, 2H), 2.73 (t, J = 9.6 Hz, 2H), 2.40 (s, 2H), 2.16 (s, 2H), 2.00 (d, J = 4.5 Hz, 12H), 1.42 (s, 9H).

^13^C NMR (101 MHz, CDCl_3_) δ = 207.16, 174.35, 172.87, 156.60, 149.97, 144.48, 139.68, 139.56, 134.05, 133.46, 132.06, 131.82, 131.03, 130.80, 130.21, 130.13, 128.11, 128.01, 127.69, 127.61, 127.13, 125.13, 124.87, 121.99, 111.23, 110.89, 102.05, 100.09, 58.09, 53.52, 51.19, 50.89, 47.78, 43.84, 42.44, 37.97, 30.93, 28.50, 27.69, 27.66, 26.47, 23.97, 20.95, 18.40.

### Synthesis of Compound 8

Compound 7 was treated with TFA/CH_2_Cl_2_ (1:20 v/v) and stirred at room temperature for 2 h, yielding a green solution. The mixture was concentrated under reduced pressure to remove volatiles, and the residue was washed with water, filtered, and purified by column chromatography (CH_2_Cl_2_/MeOH 4:1 v/v). ^1^H NMR (400 MHz, DMSO) δ8.47 (d, J = 14.3 Hz, 1H), 8.37‐8.23 (m, 3H), 8.18‐8.01 (m, 4H), 7.94 (d, J = 8.6 Hz, 1H), 7.83‐7.77 (m, 2H), 7.74 (d, J = 9.3 Hz, 1H), 7.72‐7.67 (m, 1H), 7.66‐7.61 (m, 1H), 7.57 (t, J = 8.2 Hz, 1H), 7.48 (t, J = 8.7 Hz, 1H), 6.81 (d, J = 14.4 Hz, 1H), 6.24 (d, J = 13.4 Hz, 1H), 4.66‐4.58 (m, 2H), 4.39‐4.29 (m, 2H), 2.99 (s, 2H), 2.88‐2.81 (m, 2H), 2.80‐2.72 (m, 2H), 2.63 (t, J = 6.1 Hz, 2H), 2.16‐2.01 (m, 4H), 1.98 (d, J = 7.5 Hz, 10H), 1.90 (d, J = 6.4 Hz, 3H).

^13^C NMR (101 MHz, DMSO) δ175.66, 172.09, 158.71, 147.86, 144.28, 140.99, 140.29, 139.92, 135.07, 133.12, 132.29, 131.65, 131.11, 130.80, 130.42, 130.35, 128.37, 128.17, 128.09, 127.80, 127.73, 126.53, 125.87, 125.09, 122.88, 122.59, 118.34, 115.41, 112.51, 111.75, 103.96, 99.95, 51.64, 50.70, 48.13, 43.93, 41.27, 36.98, 27.57, 27.41, 26.52, 25.82, 24.42, 21.05.

### Synthesis of Compound 9

A solution of TSTU (0.150 g, 0.5 mmol) and 3‐maleimidopropionic acid (0.085 g, 0.5 mmol) in CH_2_Cl_2_ (30 mL) was treated with Compound 8 (0.360 g, 0.5 mmol) and DIPEA (0.064 g, 0.5 mmol). The reaction mixture was stirred at room temperature for 6 h and monitored by TLC. Then eluent CH_2_Cl_2_/MeOH (15/1; v/v). ^1^H NMR (400 MHz, DMSO):δ8.35 (dd, J = 14.8, 7.2 Hz, 2H), δ8.29 (d, J = 7.5 Hz, 1H), δ8.25 (d, J = 8.5 Hz, 2H), δ8.15 (t, J = 5.8 Hz, 1H), δ8.10 ‐8.00 (m, 4H), δ7.84 (d, J = 8.8 Hz, 1H), δ7.70 (dd, J = 13.0, 8.9 Hz, 2H), δ7.62 (dd, J = 14.5, 7.7 Hz, 2H), δ7.49 (dd, J = 15.6, 8.2 Hz, 2H), δ6.60 (d, J = 14.3 Hz, 1H), δ6.38 (t, J = 14.3 Hz, 2H), δ4.54‐4.47 (m, 2H), δ4.39‐4.30 (m, 4H), δ3.47 (d, J = 5.4 Hz, 2H), δ3.40 (dd, J = 7.0, 4.9 Hz, 2H), δ2.75 (dd, J = 25.8, 6.1 Hz, 4H), δ2.58 (t, J = 6.6 Hz, 2H), δ2.10‐2.02 (m, 2H), δ1.92 (s, 10H), δ1.87‐1.81 (m, 2H), δ1.59 (s, 3H).

^13^C NMR (126 MHz, DMSO‐d6):δ174.03, 173.68, 173.54, 169.93, 147.10, 142.45, 141.79, 141.41, 139.84, 139.75, 139.60, 133.72, 133.42, 133.18, 131.43, 131.34, 131.25, 130.40, 130.06, 129.80, 127.67, 127.60, 127.37, 127.33, 126.56, 126.02, 124.95, 124.74, 122.14, 111.77, 111.45, 101.96, 101.25, 100.83, 54.82, 50.67, 50.53, 48.47, 47.59, 43.49, 43.38, 43.01, 36.58, 26.90, 26.70, 25.92, 25.82, 23.69, 22.33, 20.48.

### Synthesis of Compound 10

A solution of cystamine dihydrochloride (0.115 g, 0.5 mmol) in CH_2_Cl_2_ (30 mL) was treated with Compound 10 (0.435 g, 0.5 mmol) and TEA (0.01 g, 0.1 mmol). The mixture was stirred for 6 h at room temperature, monitored by TLC, and purified by column chromatography (CH_2_Cl_2_/MeOH 15:1 v/v) to afford the product as a blue solid.

^1^H NMR (400 MHz, DMSO‐d6):δ8.43‐8.34 (m, 1H), δ8.33‐8.28 (m, 1H), δ8.18‐8.12 (m, 2H), δ8.11 ‐8.05 (m, 2H), δ7.98 (d, J = 8.7 Hz, 3H), δ7.90‐7.84 (m, 1H), δ7.73 (d, J = 8.9 Hz, 1H), δ7.66 (d, J = 8.9 Hz, 2H), δ7.61‐7.55 (m, 2H), δ7.53 (d, J = 8.9 Hz, 1H), δ7.40 (t, J = 7.4 Hz, 2H), δ6.64 (d, J = 14.2 Hz, 1H), δ6.41 (d, J = 14.6 Hz, 1H), δ5.97 (d, J = 13.6 Hz, 2H), δ4.58‐4.52 (m, 1H), δ4.41‐4.37 (m, 1H), δ4.29 (s, 2H), δ4.19‐4.06 (m, 4H), δ3.17 (s, 4H), δ3.07 (d, J = 10.1 Hz, 2H), δ2.94 (dd, J = 12.3, 5.9 Hz, 2H), δ2.84‐2.75 (m, 2H), δ2.61 (dd, J = 14.7, 5.2 Hz, 7H), δ2.08‐2.03 (m, 2H), δ1.97 (s, 4H), δ1.93 (s, 9H), δ1.81‐1.75 (m, 3H), δ1.68 (s, 1H), δ1.63 (s, 1H).

^13^C NMR (126 MHz, DMSO‐d6):δ173.63, 169.84, 168.58, 147.10, 142.43, 141.44, 140.50, 139.83, 139.61, 138.02, 133.69, 133.21, 131.34, 130.76, 130.25, 129.71, 127.77, 127.17, 126.54, 125.97, 124.83, 123.41, 122.15, 121.62, 120.11, 111.76, 110.83, 101.89, 100.90, 94.58, 69.65, 50.65, 49.03, 48.46, 48.04, 47.60, 45.16, 37.61, 36.44, 35.87, 33.74, 27.73, 27.52, 26.90, 26.70, 25.92, 24.62, 23.67, 22.87, 22.43, 22.33, 21.40, 20.48, 8.29.

### Synthesis of CYMMAF

The cytotoxic drugs MMAF (10 mg, 0.014 mmol) and EDC·HCl (2.62 mg, 0.014 mmol) were dissolved in anhydrous DMF, stirred at room temperature for 1 h, and then added near‐infrared fluorescent dye (20 mg, 0.2 mmol) (Compound 10). CYMMAF was isolated by column chromatography at room temperature with eluent MeOH/CH_2_Cl_2_ (5/1; v/v).

^1^H NMR (400 MHz, DMSO) δ 8.38 – 8.29 (m, 2H), 8.25 (dd, J = 15.4, 7.1 Hz, 2H), 8.14 ‐8.07 (m, 3H), 8.07 – 7.99 (m, 3H), 7.93 (d, J = 9.0 Hz, 3H), 7.84 (d, J = 6.9 Hz, 1H), 7.70 (d, J = 8.4 Hz, 1H), 7.61 (d, J = 9.2 Hz, 3H), 7.56 – 7.44 (m, 4H), 7.38 – 7.32 (m, 2H), 6.58 (d, J = 14.2 Hz, 1H), 6.37 (d, J = 13.8 Hz, 1H), 6.01 – 5.90 (m, 2H), 5.30 – 5.26 (m, 1H), 4.53 – 4.46 (m, 1H), 4.35 (s, 1H), 4.24 (s, 3H), 4.10 (dd, J = 10.2, 5.0 Hz, 3H), 4.04 (s, 2H), 3.99 – 3.94 (m, 1H), 3.78 (s, 1H), 3.47 (s, 3H), 3.13 (d, J = 5.2 Hz, 6H), 3.02 (s, 2H), 2.91 (d, J = 6.2 Hz, 3H), 2.75 (d, J = 16.4 Hz, 4H), 2.58 (d, J = 5.1 Hz, 3H), 2.54 (s, 4H), 2.17 – 2.11 (m, 2H), 2.02 – 1.81 (m, 23H), 1.74 (s, 4H), 1.64 (s, 2H), 1.58 (s, 2H), 1.29 (s, 2H), 1.19 (s, 15H), 0.81 (t, J = 6.8 Hz, 3H).

^13^C NMR (126 MHz, DMSO) δ 174.95, 174.78, 174.24, 174.15, 170.51, 147.69, 143.01, 142.02, 141.05, 140.42, 140.19, 134.30, 133.78, 132.01, 131.84, 131.81, 130.99, 130.84, 130.64, 130.58, 130.38, 130.31, 130.13, 130.11, 128.35, 128.23, 128.19, 127.96, 127.92, 127.77, 127.67, 127.14, 126.61, 125.53, 125.33, 124.01, 122.77, 122.22, 114.28, 112.36, 112.05, 111.42, 102.52, 101.45, 70.24, 55.92, 51.25, 51.13, 49.61, 49.05, 48.63, 48.18, 38.28, 38.19, 37.03, 35.58, 34.89, 34.61, 34.13, 31.76, 30.83, 29.55, 29.54, 29.50, 29.46, 29.38, 29.34, 29.30, 29.21, 29.17, 29.06, 29.05, 29.01, 28.95, 28.32, 28.25, 28.10, 27.49, 27.29, 27.03, 26.51, 26.41, 25.59, 24.95, 24.27, 24.19, 23.47, 23.02, 22.92, 22.57, 21.98, 14.44, 14.04.

### Synthesis of UdAb n501—Synthesis and Characterization of n501‐CYMMAF

The anti‐5T4 antibody n501 was treated with TCEP (1 mm) at a 3:1 molar ratio (TCEP: n501) for 1 h at 4 °C to reduce disulfide bonds. CYMMAF (2 mg), pre‐dissolved in DMSO/MeCN (100 µL/500 µL), was added batchwise to the reaction mixture, followed by vortex mixing and incubation at 4 °C for 1 h. Unreacted CYMMAF was removed using a Zeba desalting column. The n501‐CYMMAF conjugate was centrifuged in a gradient (4000, 10 000, and 12 000 rpm, 5 min per step), and the blue supernatant was collected. Purity was verified by HPLC and RC‐MS, while binding affinity was assessed via ELISA.

### Optical Spectra Measurement

CYMMAF (10 µm) in EtOH/PBS (1:4 v/v) was prepared for UV–vis and fluorescence spectral analysis. Cysteine (280 µm) was added to mimic the intracellular microenvironment. To evaluate the ratiometric probe potential of CYMMAF, pH‐dependent UV–vis spectral changes were measured using buffers (pH 2–10) prepared with K2HPO4/KH2PO4 (0.1 mol L^−1^), HCl, and NaOH. For microenvironment simulation, CYMMAF (10 µm) in pH 5 (acidic tumor‐like) and pH 7.4 (blood‐like) buffers was monitored by UV–vis and fluorescence spectroscopy at 10‐min and 1‐h intervals, respectively. Fluorescence spectra were acquired using an FLS920 spectrometer with a 630‐nm excitation source and 680‐nm longpass filter.

### ELISA Characterization

The 5T4 antigen was immobilized on Costar half‐area high‐binding ELISA plates at 100 ng per well through overnight incubation (4 °C) in phosphate‐buffered saline (PBS). Subsequently, plates were blocked with 3% (w·v^−1^) skim milk powder solution for 1 h at 37 °C. Serial dilutions of three experimental groups – CYMMAF control, TCEP‐free n501, and n501 supplemented with 1 mm TCEP (Sigma–Aldrich) – along with n501‐CYMMAF complexes were incubated in triplicate wells for 1.5 h at 37 °C. Antigen‐specific binding was detected using a monoclonal anti‐Flag horseradish peroxidase (HRP)‐conjugated antibody (Clone M2, Sigma–Aldrich, Cat# A8592) with 1:5000 dilution. Enzymatic activity was quantified by measuring the absorbance at 405 nm (OD405) using a BioTek Synergy H1 microplate reader following 10‐min substrate development with 2,2′‐azino‐bis (3‐ethylbenzothiazoline‐6‐sulfonic acid) diammonium salt (ABTS; Thermo Fisher Scientific).

Anti‐MMAF monoclonal antibody (MAb; 11b8, Mouse, GENEWIZ Inc.) was used to calibrate the fluorescence ratio versus cleavage efficiency. Briefly, 2 ng well^−1^ of anti‐MMAF MAb was coated on a 96‐well ELISA plate and incubated overnight, followed by blocking with 3% BSA for 1 h and washing three times with PBST using a BioTek 405 LS Washer. Serially diluted n501‐CYMMAF (initial concentration: 100 µg; three‐step dilution) was prepared in 1% BSA for quantification of MMAF content. Samples of CYMMAF (100 µg) preincubated at pH 5.5 or pH 7.4 and 37 °C for different times (0–24 h) were added to the plate and incubated for 1.5 h, followed by three washes. Anti‐FLAG HRP (Rabbit mAb; Art. No. S‐589‐8) was then added for 45 min, followed by five washes. Finally, 50 µL well^−1^ of ABTS substrate (Thermo Fisher, Art. No. 002024) was added and incubated for 10 min at 37 °C before absorbance measurement at 405 nm using a Tecan microplate reader.

### Mass Spectra Characterization

To characterize n501‐CYMMAF, two complementary mass spectrometry techniques were used: RC‐MS and LC‐MALDI‐TOF‐MS. Each method was chosen for its specific analytical strength. LC‐MALDI‐TOF‐MS was applied to study the dissociation behavior of the conjugate because its mobile phase (acetonitrile with TFA) can promote the release of small molecules^[^ ^36^, ^37^S. ^]^. This feature makes it suitable for detecting and identifying dissociated byproducts, which was important for understanding the degradation pathway. In contrast, RC‐MS was a gentler and more biocompatible method that confirmed the intact nanobody‐dye conjugate without causing significant dissociation.

Both techniques provided useful information, but a small difference in molecular weight was observed. This was likely due to variations in ionization and solvent conditions. For mechanistic interpretation, the focus was placed on LC‐MALDI‐TOF‐MS results, since they directly reflected the dissociation process related to the proposed degradation mechanism. This approach ensures that the conclusions are consistent with the experimental evidence and support the reliability of the model.

### Immunofluorescent Imaging

SKOV3/luc cells (1 × 10⁵ well^−1^; RRID: CVCL_5J38) were plated in 24‐well plates and cultured overnight. Cells were treated with 400 nm n501 or n501‐CYMMAF (prepared in PBS to enhance uptake) for 0.5, 1 h, 1.5, or 2 h at 37 °C, for the last 30 mins, Lyso‐Tracker Green was added to the medium to label the lysosome. Following PBS washes (3 × 1 mL), cells were fixed with 4% paraformaldehyde (RT, 5 min) and stained with 5 µg mL^−1^ DAPI (10 min). After three PBS washes, slides were mounted and imaged using a Leica microscope. DAPI (λ_em_ = 420 nm), Lyso‐Tracker Green (λ_em_ = 590 nm), and red fluorescence (λ_em_ = 650–730 nm) signals were analyzed.

Ex vivo cytotoxicity of n501‐CYMMAF and n501: 5T4‐expressing SKOV3 cells (RRID: CVCL_5J38), non‐tumor cell lines HMEC‐1 (human origin; RRID: CVCL_0307) and CHO (hamster origin; RRID: CVCL_0213), and the endothelial cell line bEND.3 (RRID: CVCL_0170) were used for targeted toxicity analysis (Figure 4A–D). SKOV3‐luc, HMEC‐1, CHO, and bEND.3 cells were seeded into a 96‐well cell culture plate (5000 cells per well) and incubated for 12 h. After discarding the culture supernatant, 100 µL of n501, irrelevant sdAb‐CYMMAF, or n501‐CYMMAF solutions were added, each diluted in a gradient starting from an initial concentration of 1 µm, with triplicate repeats for each condition. The cells were cultured for 48 h at 37 °C before adding 100 µL of CCK‐8 solution per well (10 µL CCK‐8 + 90 µL medium). Absorbance was measured at 405 nm after a 3‐h incubation using a 96‐well microplate reader. The cell viability was calculated using the following formula:

(1) $$\begin{array}{ccc} \mathrm{Cell}\;\mathrm{viability}\;\left(\%\right) & = & \left[\left(OD_{\mathrm{experimental}\;\mathrm{group}}-OD_{\mathrm{medium}}\right)\right.\\ & & /\left.\left(OD_{\mathrm{medium}}-\;OD_{\mathrm{reference}\;\mathrm{well}}\right)\right]\times 100\% \end{array}$$

### Flow Cytometry Experiment

To evaluate whether the ADC drug can bind to tumor cells, SKOV3 cells (1 × 10⁶ per tube) were collected and incubated with 250 or 500 nm n501‐MMAF antibody diluted in flow staining buffer at 4 °C for 20 min. After incubation, 2 mL of FBS‐containing wash buffer was added to resuspend the cells, followed by centrifugation at 250 × g for 5 min. The washing step was repeated twice. The cells were then resuspended in PBS, and antibody binding was analyzed using a flow cytometer in the APC channel.

### Pharmacokinetic Monitoring of n501‐CYMMAF in Mice

Mice aged 6–8 weeks were intraperitoneally injected with 100 µg of n501‐MMAF per mouse. Blood samples were collected using anticoagulant tubes at 15 min, 30 min, 1 h, 2 h, 4 h, 6 h, 8 h, 12 h, and 24 h post‐administration. The samples were centrifuged at 3000 rpm at 4 °C for 10 min to separate plasma, and the supernatant was collected. The plasma concentration of the antibody was then quantified using an ELISA assay.

For the quantification of antibody concentration by ELISA, 2 ng well^−1^ of anti‐MMAF MAb was coated on a 96‐well ELISA plate and incubated overnight, followed by blocking with 5% BSA for 1 h and washing three times with 0.05% PBST. 50 µL of three‐fold serially diluted antibody in PBS or diluted plasma was added for binding at 37 °C for 1.5 h, Secondary antibody, anti‐Flag‐HRP, was added accordingly for another 45 min at 37 °C. The plate was washed with PBST for five times, and the enzyme activity was measured by recording the absorbance at 405 nm after incubation with ABTS substrate (Invitrogen). 12‐gradient serially diluted purified antibodies were used to generate a quantitative standard curve. Specifically, the optical densities (OD) of a set of antibody concentration standards were determined and used to plot an OD versus concentration standard curve that was analyzed by a four‐parameter curve fit. The serum concentrations of the antibody were calculated from the standard curve.

### Diagnosis and Treatment Experiment

Bioluminescence and fluorescence imaging were performed weekly using an IVIS Spectrum system (PerkinElmer) in an SPF‐certified animal facility. SKOV3/luc cells were purchased from Fuheng Biology (FH0009). The cells were collected by centrifugation at 800 rpm, resuspended in sterile PBS, and intraperitoneally injected in two separate batches (1 × 10⁶ cells per mouse) into a female nude mouse (6 weeks old) each time. After 14 days, tumor growth was monitored by intraperitoneal injection of 200 µL D‐luciferin (15 mg mL^−1^), with imaging taken 10 min post‐injection. Then the mice were divided into two groups randomly, accepting intraperitoneal injection of PBS or n501‐CYMMAF (1 mg mL^−1^, 100 µL per mouse) two times per week. Then after four‐weeks of treatment, the mice accepted bioluminescent bioimaging and NIR bioimaging ((λ_em_ = 630–660 nm and λ_em_ = 730–750 nm) for comparison. Mice were euthanized using isoflurane anesthesia followed by cervical dislocation to ensure complete death, after which anatomical analysis was performed. Excised tumors and suspected tissues were collected for immunofluorescence and H&E staining.

### Immunofluorescent Imaging of Tumor Tissue

Slides were placed in a 60 °C oven for 10 min, followed by deparaffinization and rehydration: slides were immersed sequentially in xylene, 100% ethanol, 95% ethanol, and 70% ethanol. Afterward, the slides were washed with 0.1% TBST three times, each for 5 min. Antigen retrieval was performed by placing the slides in preheated antigen retrieval buffer at 65 °C, followed by steaming at 97 °C for 30 min. Blocking was performed with 5% donkey serum at room temperature for 30 min, followed by three washes with 0.1% TBST. The slides were then incubated overnight at 4 °C with the primary antibody. The next day, after three washes with 0.1% TBST, the slides were incubated in the dark at room temperature for 2 h with secondary antibody, followed by three washes with 0.1% TBST. All subsequent steps were also performed in the dark. The nuclei were stained with DAPI diluted 1:2000 for 5 min at room temperature, followed by three TBST washes. After removing excess liquid, antifade mounting medium was applied to seal the coverslip.

### Statistical Analysis

The gel shown in Figure 2J was run as three adjacent lanes on the same gel for the marker, n501, and n501‐CYMMAF, without any splicing or further editing. TBR values were analyzed using Bruker MI SE software (n = 3), with error bars representing mean ± variance (Figure 5). ELISA (n = 3) (Figure 3E), CCK‐8 cytotoxicity assays (n = 3) (Figure 3A–D), and body weight changes (n = 5) were processed using GraphPad Prism 8 (Figure 5E). Metastasis treatment experiments included five mice per group. Ratiometric fluorescence (633/800 nm) was calculated in Bruker MI SE based on pixel division of the imaging data. Abdominal total flux was quantified using one‐way ANOVA with Bonferroni correction (Figure 5E). The fluorescent spectrometer for Figure S11 (Supporting Information) was collected on FS5 (Edinburgh) without filter and that for Figures 2E–G and 3A,G was FLS920 with a 680‐nm longpass filter.

## Conflict of Interest

The authors declare no conflict of interest.

## Supporting information



Supporting Information

## Data Availability

The data that support the findings of this study are available from the corresponding author upon reasonable request.
