# Supplementary material for: NIR Ratiometric Fluorescent Antibody‐Drug Conjugate for Metastatic Ovarian Cancer Theranostics and Treatment Response Monitoring
Source: Adv Sci (Weinh). 2025 Nov 28;13(7):e16607. doi: 10.1002/advs.202516607 (PMC12866695; doi:10.1002/advs.202516607)
Supplement: Supplementary file 1 — Supporting Information [file ADVS-13-e16607-s001.docx]

**NIR Ratiometric Fluorescent Antibody-Drug Conjugate for Metastatic Ovarian Cancer Theranostics and Treatment Response Monitoring**

*Cheng Li^1a, d^, Zezhong Yu^1b, c,^ Tao Pu^1a^, Yanling Wu^1a, d^, Gang Wang^a^, Youhua Xie*^a^, Tianlei Ying*^a, b^, Zhenlin Yang^*a, b, c^, Yibing Shi*^a^.*

C. Li, Z. Z. Yu, T. Pu, Y.L. Wu, G. Wang, Z.L. Yang, Y. H. Xie, T.L. Ying, Y.B. Shi,

Shanghai Institute of Infectious Disease and Biosecurity, Institute for brain translational research, Obstetrics & Gynecology Hospital of Fudan University, School of Basic Medical Sciences, Fudan University. Shanghai 200032, China.

Email: [shiyibing@fudan.edu.cn;](mailto:shiyibing@fudan.edu.cn;) [yang_zhenlin@fudan.edu.cn;](mailto:yang_zhenlin@fudan.edu.cn;) [tlying@fudan.edu.cn](mailto:yintianlei@fudan.edu.cn);

[yhxie@fudan.edu.cn](mailto:yhxie@fudan.edu.cn).

[b] Z.Z. Yu, Z.L. Yang, Department of Pulmonary Medicine, Zhongshan Hospital, Fudan University, Shanghai 200032, China

[c] Z.Z. Yu, Z.L. Yang, Shanghai Key Laboratory of Lung Inflammation and Injury, Shanghai 200032, China

[d] C. Li, Y.L. Wu, T.L. Ying, Z.L. Yang, Shanghai Engineering Research Center for Synthetic Immunology, Shanghai 200032, China

^1^The authors equally contributed to this work.

Keywords: Near infrared bioimaging; Ratiometric probe; Fluorescent antibody-drug conjugate; Ovarian metastases treatment; in-situ treatment response monitoring


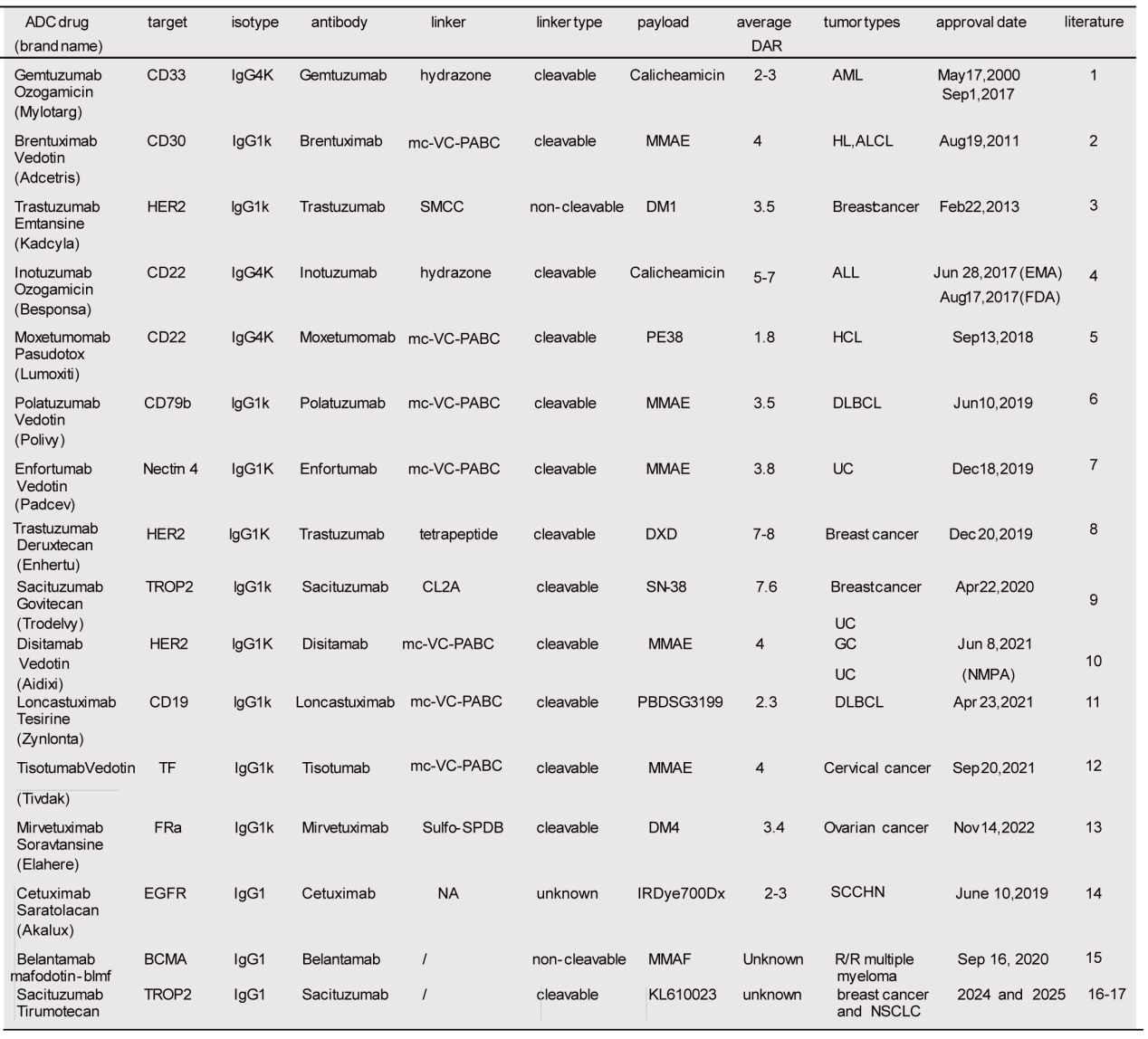


Figure S1. Summary of global clinically approved ADC drugs^1-17^


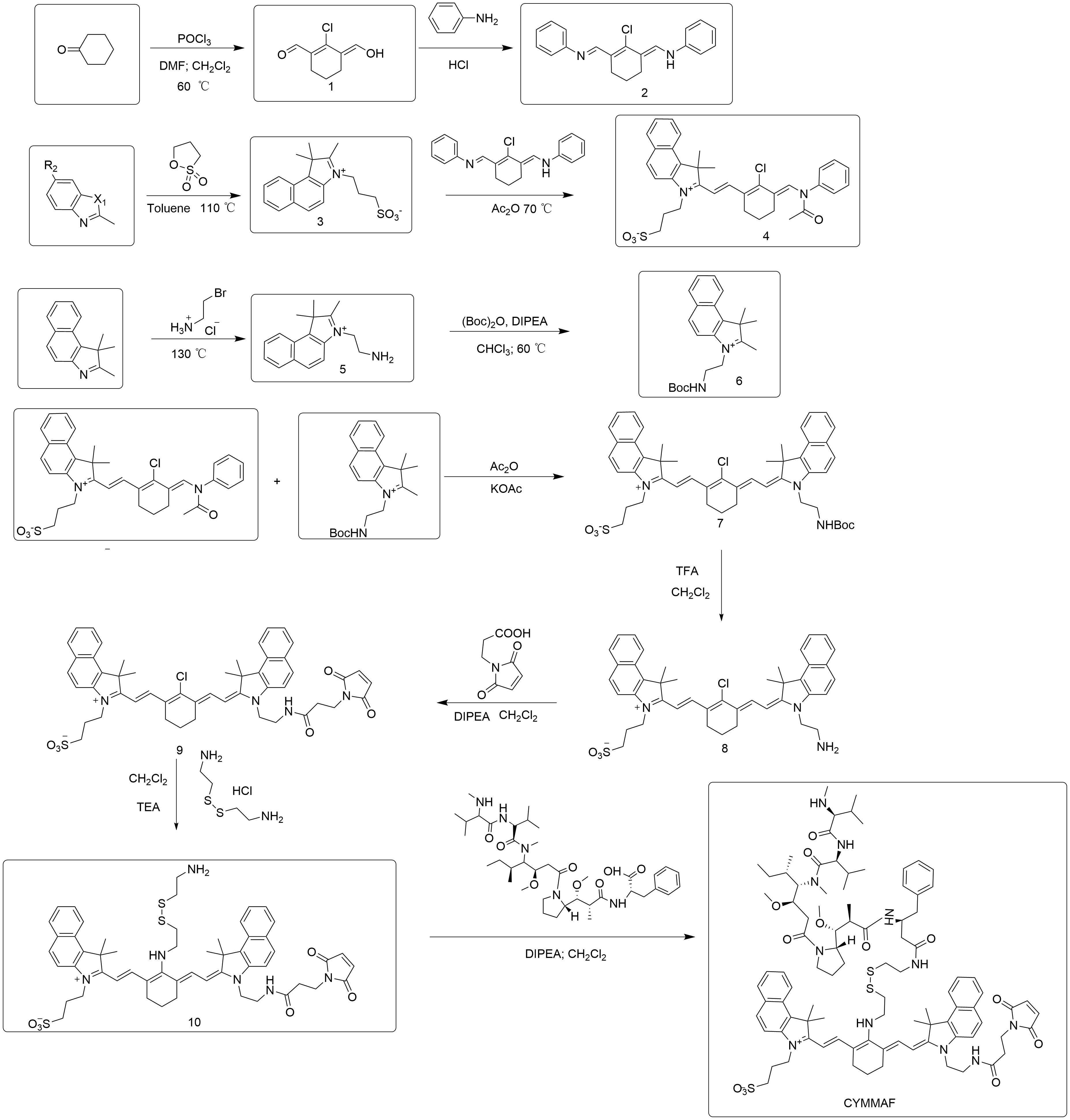


Figure S2. Synthesis of CYMMAF


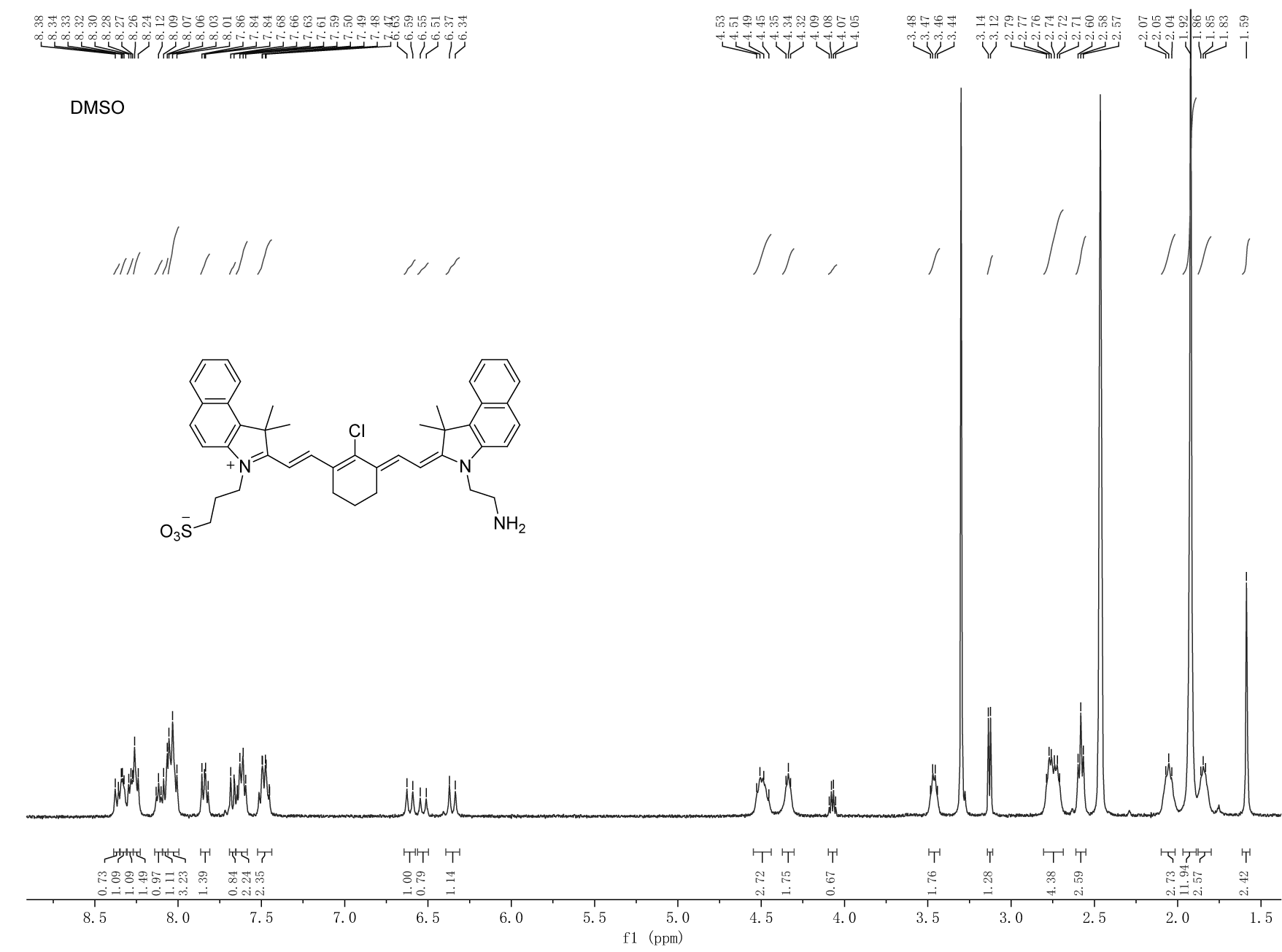


Figure S3. ^1^H spectrum of Compound 8.


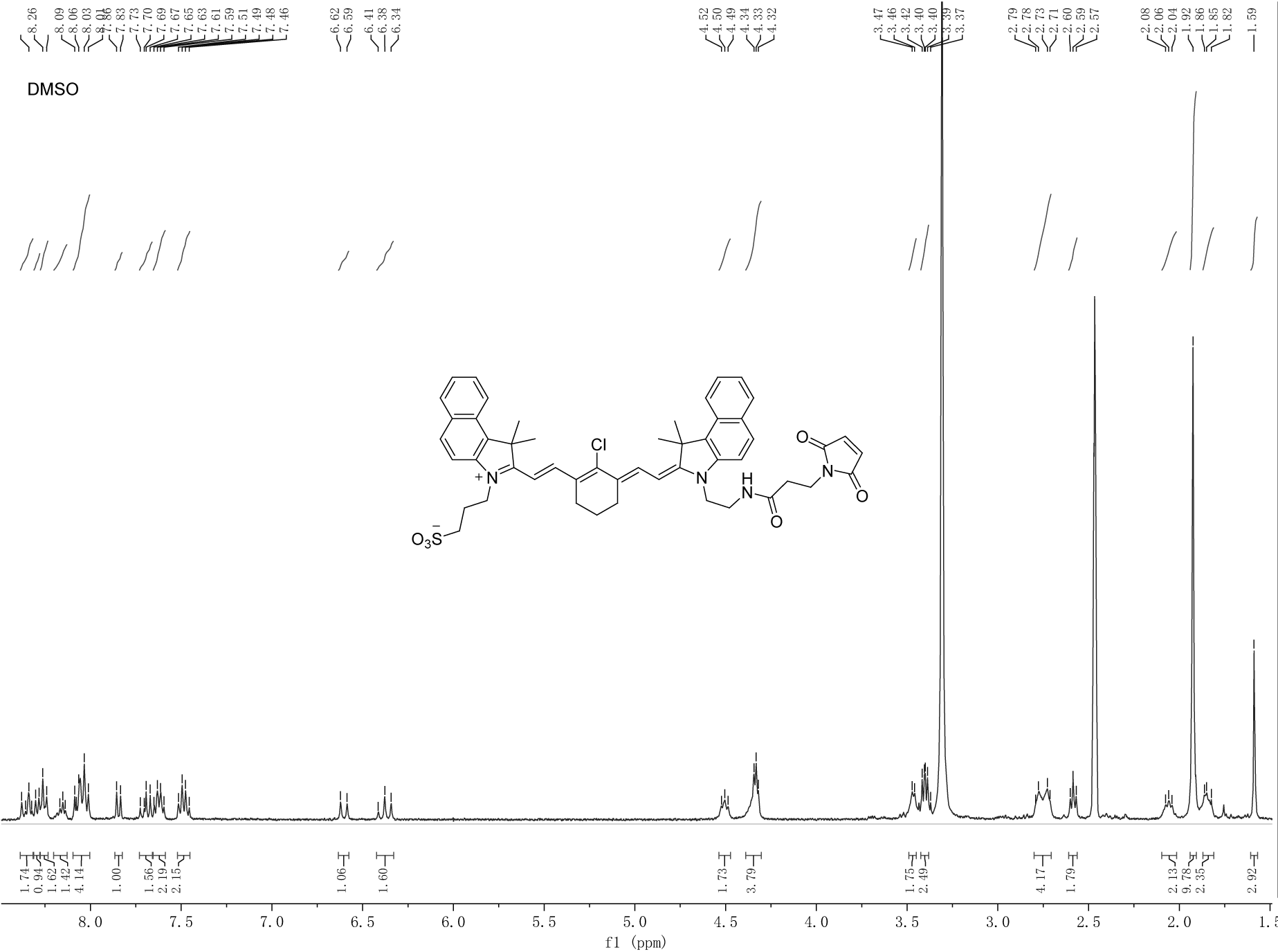


Figure S4. ^1^H spectrum of Compound 9.


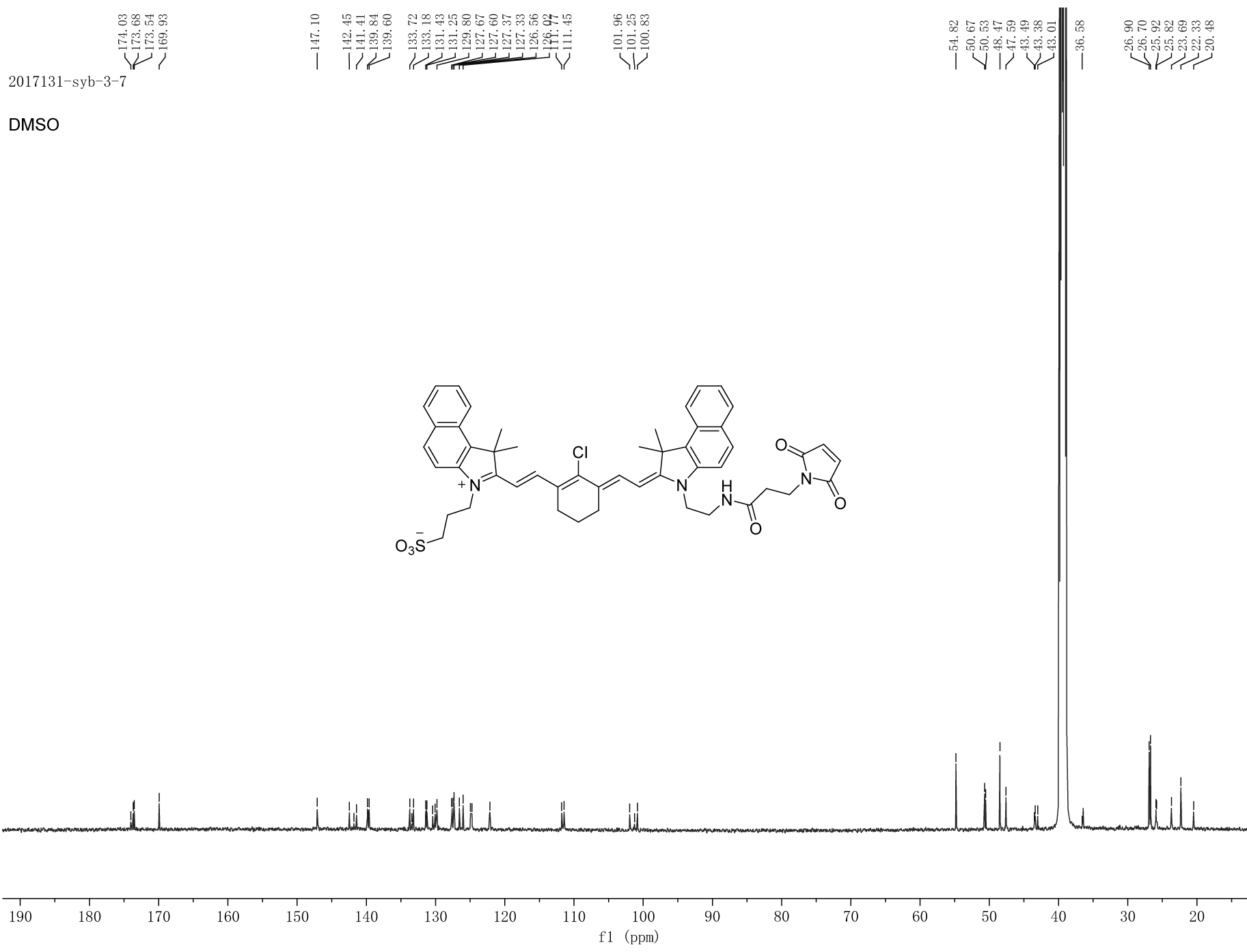


Figure S5. ^13^C spectrum of Compound 9.


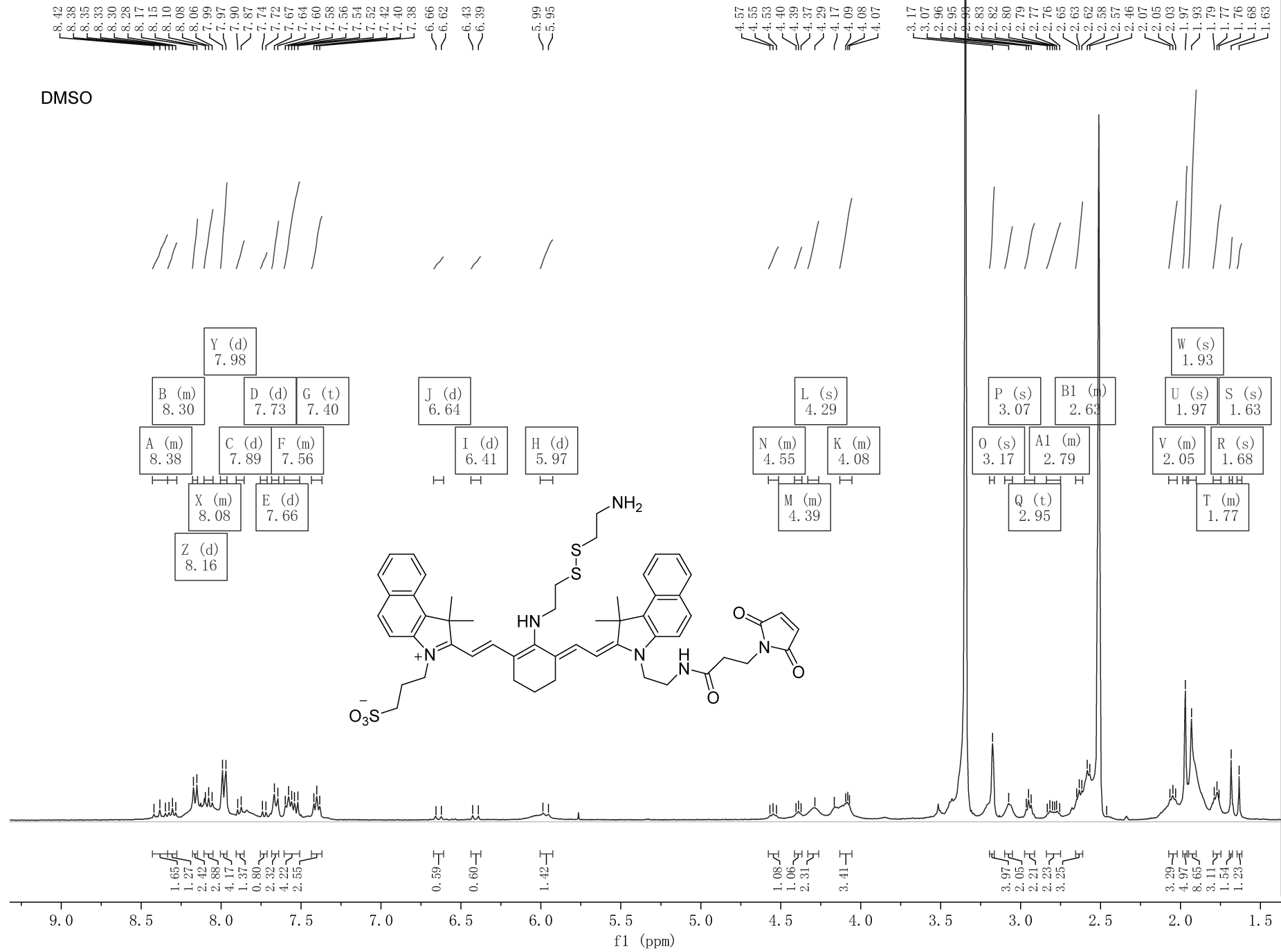


Figure S6. ^1^H spectrum of Compound 10.


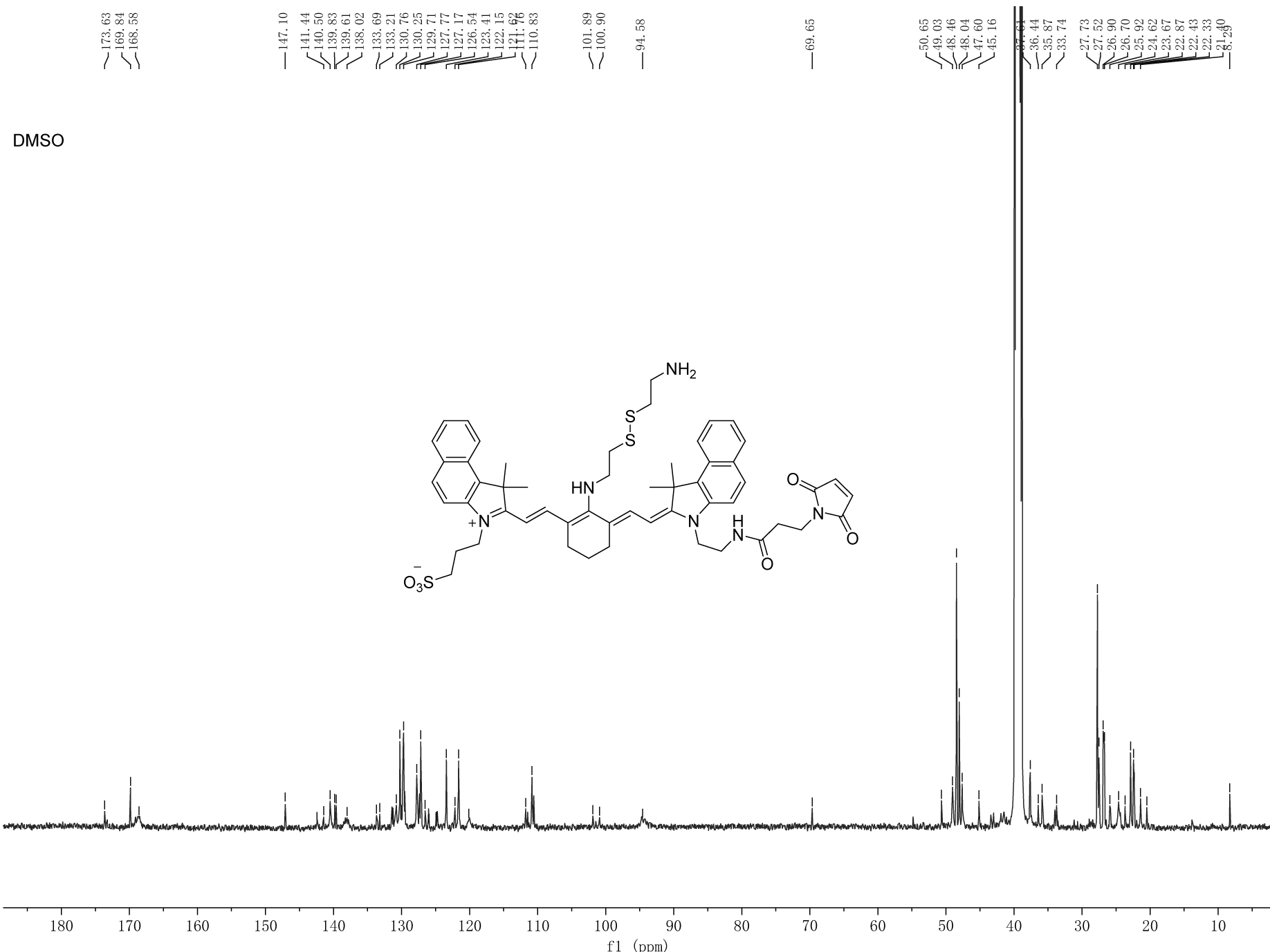


Figure S7. ^13^C spectrum of Compound 10.


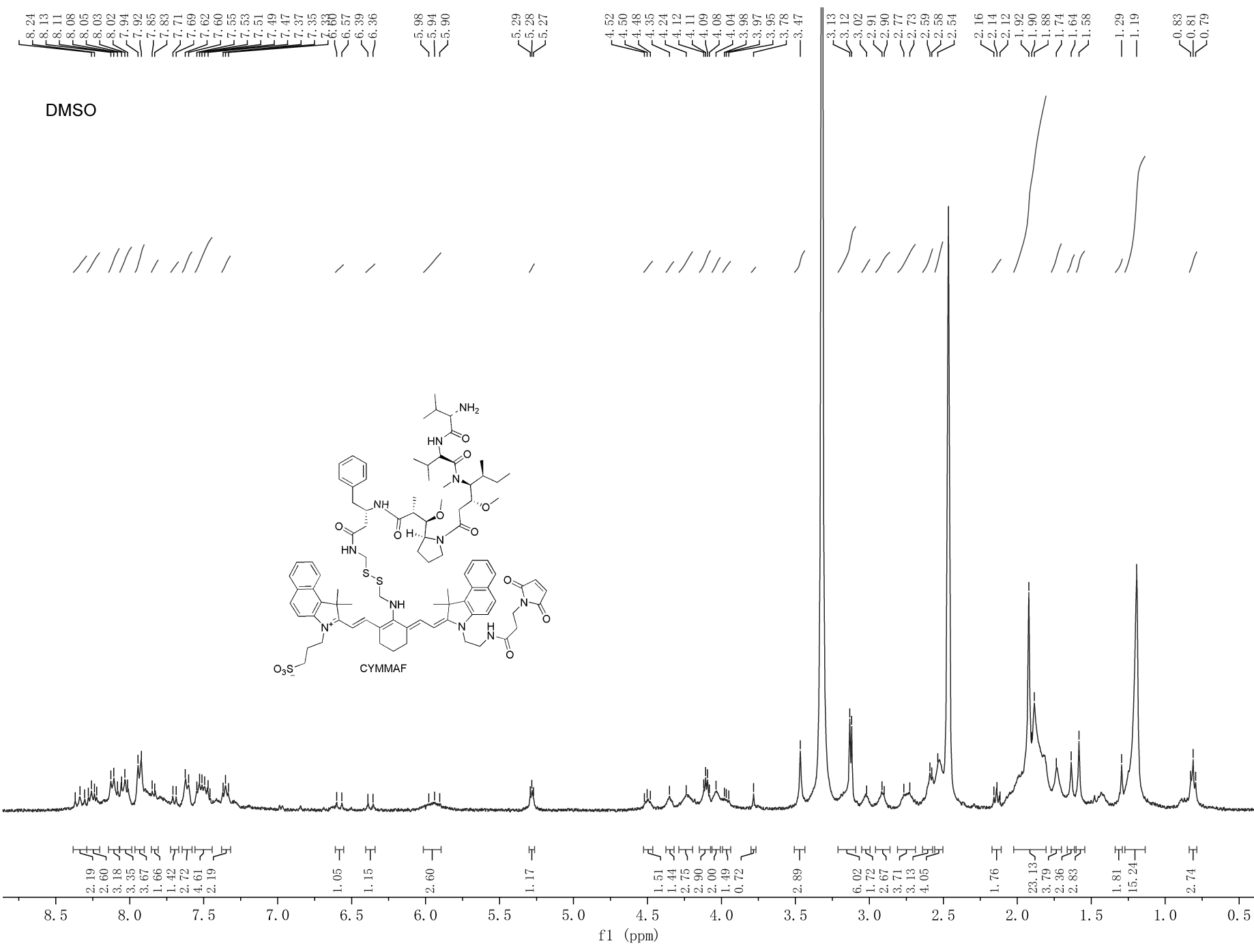


Figure S8. ^1^H spectrum of CYMMAF.


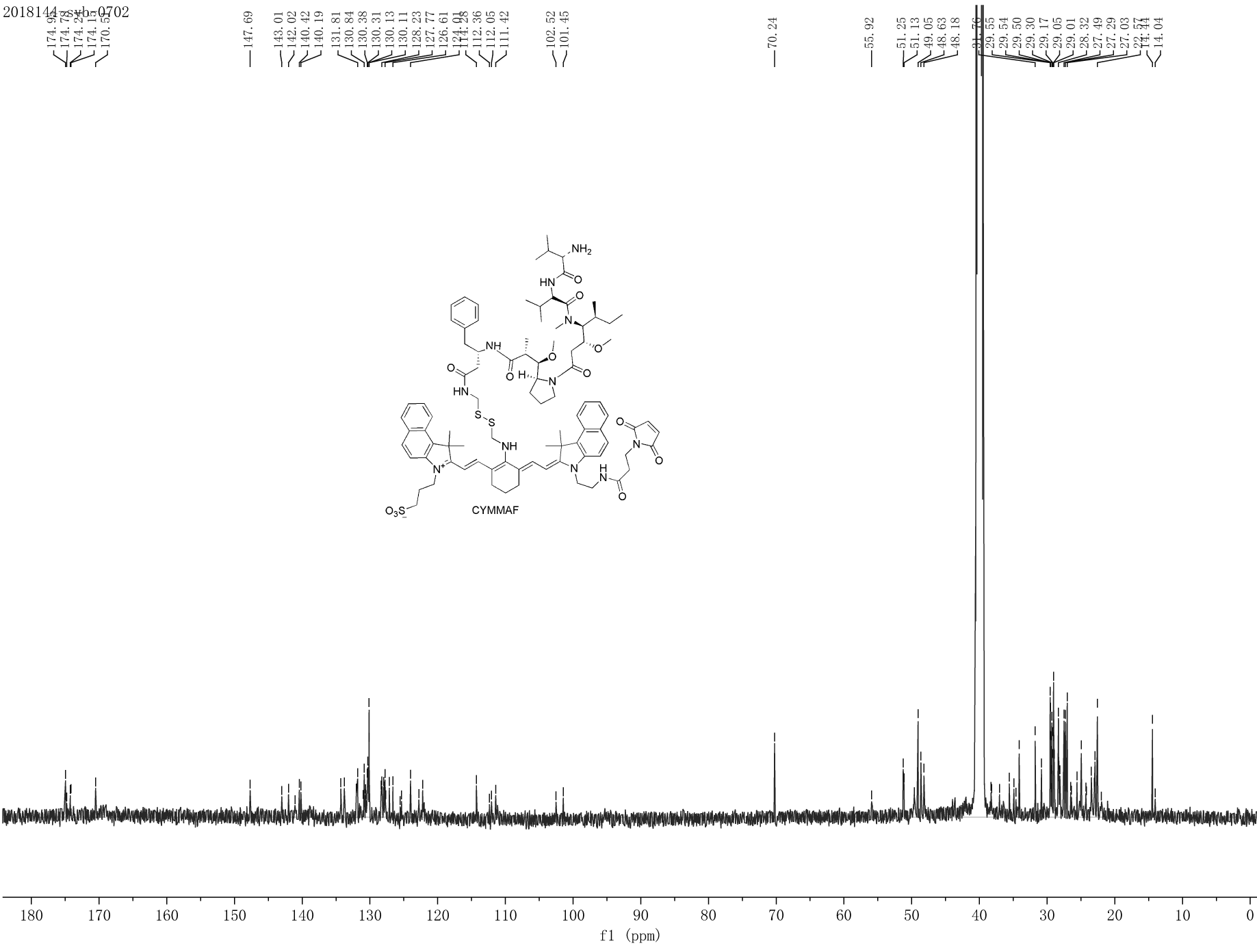


Figure S9. ^13^C spectrum of CYMMAF.


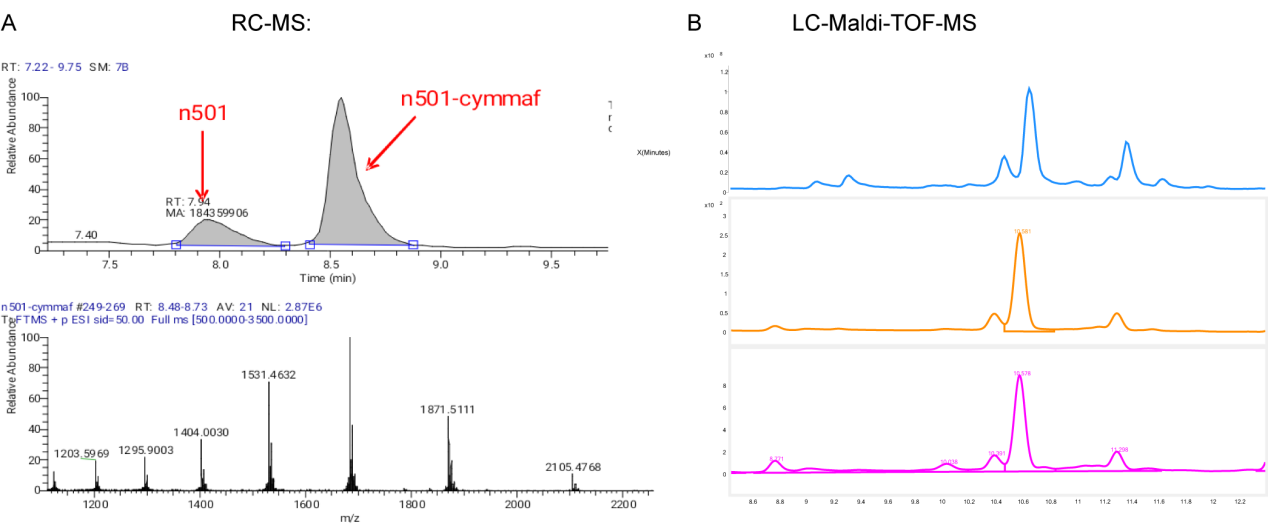


Figure S10. Original data of RC-MS (Biotree Company) and LC-Maldi-TOF-MS.

In our comprehensive characterization of n501-CYMMAF, we employed two complementary mass spectrometry techniques (RC-MS and LC-MALDI-TOF-MS) each selected for its distinct analytical advantages in elucidating different aspects of the system. Specifically, LC-MALDI-TOF-MS was deliberately chosen to investigate the dissociation behavior of the conjugate, as its mobile phase conditions (acetonitrile containing TFA) are known to promote the release of small molecules^18-19^. This property makes it particularly effective for detecting and identifying dissociated byproducts, which is critical for elucidating the degradation pathway of this conjugate. In contrast, RC-MS, a gentler and more biocompatible technique, enabled the verification of the intact n501-CYMMAF without inducing substantial dissociation, thereby complementing the LC-MALDI-TOF-MS analysis.

While both methods provided meaningful insights, a slight discrepancy in the calculated molecular weights was observed, likely due to differences in ionization behavior and solvent environments between the two techniques. To ensure mechanistic consistency and relevance, we prioritized LC-MALDI-TOF-MS data as the principal basis for mechanistic interpretation, given that it directly captures the dissociation process central to our proposed degradation mechanism. This methodological choice ensures that our conclusions are grounded in experimental conditions that accurately reflect the degradation behavior, thereby strengthening the reliability and coherence of our mechanistic model.


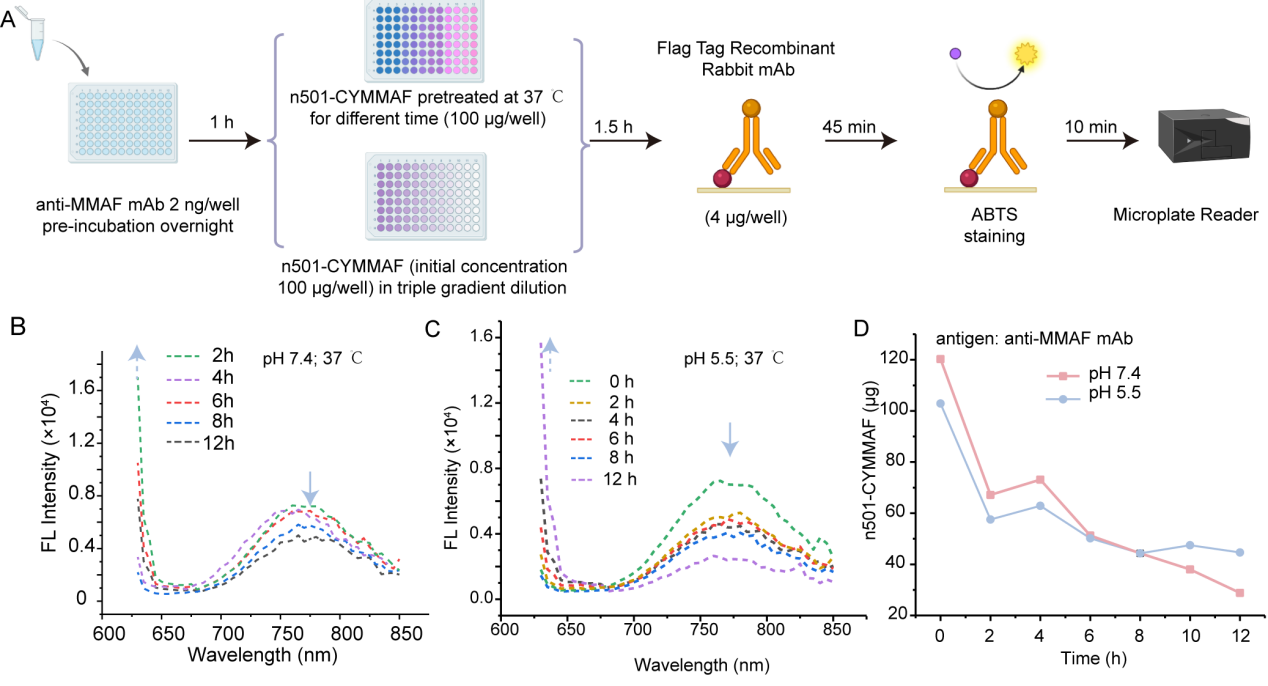


Figure S11. Calibration of fluorescence ratio versus cleavage efficiency. (A) Workflow for MMAF detection and cleavage quantification of n501-CYMMAF. (B) Fluorescence spectra of n501-CYMMAF (0.05 mg/mL) at pH 7.4, 37 °C over 0-12 h. (C) Fluorescence spectra of n501-CYMMAF (0.05 mg/mL) at pH 5.5, 37 °C over 0-12 h. (D) Quantification of CYMMAF (100 µg) after incubation at pH 5.5 or 7.4, 37 °C for 0-12 h. Fluorescent spectrometer here was Edinburgh FS5 spectrometer without the 630-nm filter and the excited source was Xe lamp (slit width: 5.0).


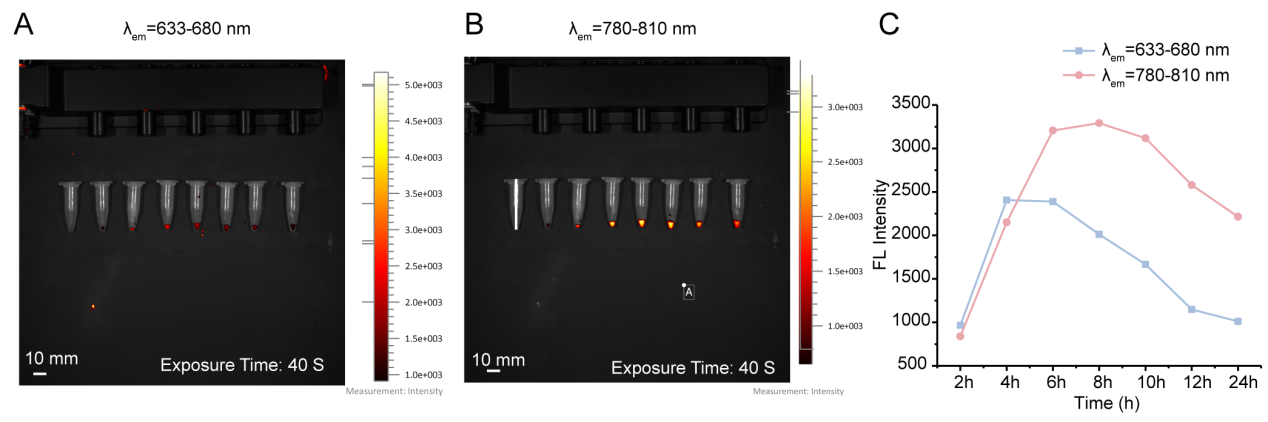


Figure S12. NIR bioimaging of blood samples (0-24 h). (A) NIR bioimaging of blood samples (0-24 h) at λ_em_ = 633-680 nm, (B) NIR bioimaging of blood samples (0-24 h) at λ_em_ = 780-810 nm, and (C) average fluorescent intensity of blood samples at different time (0-24 h).

Figure S13. Liver and kidney function test indicators and complete blood count results (100 µg / mouse, intravenous injection)..


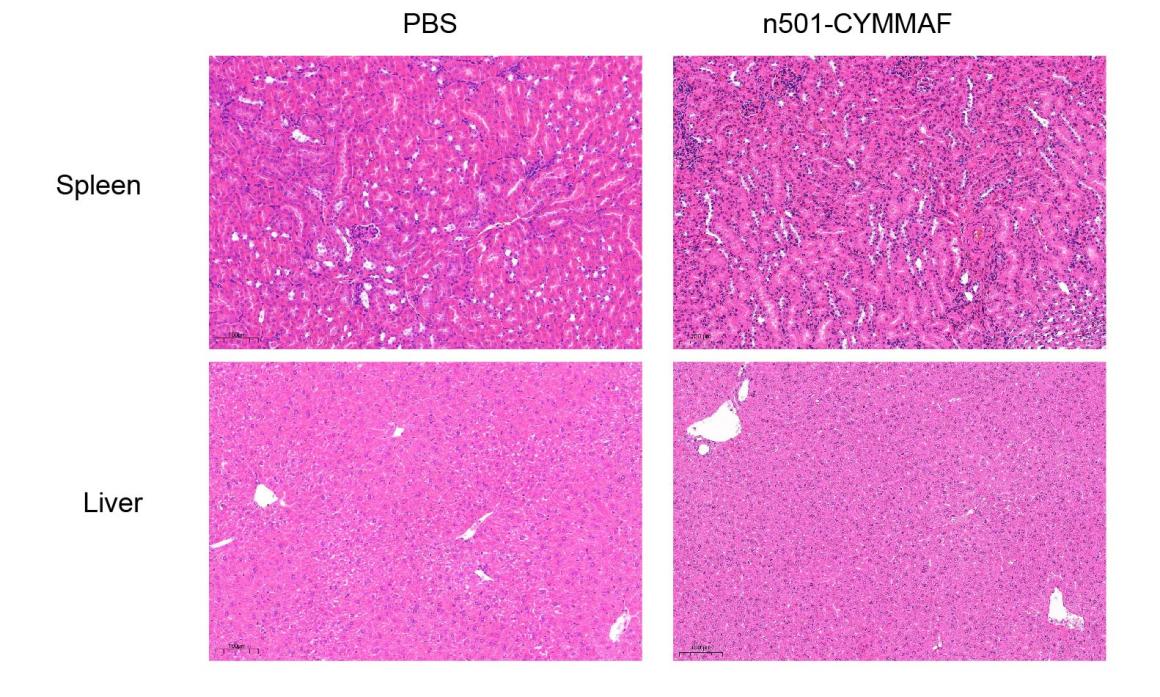


Figure S14. H&E analysis of mice spleen and liver after accepting PBS and n501-CYMMAF (100 µg) through intravenous injection


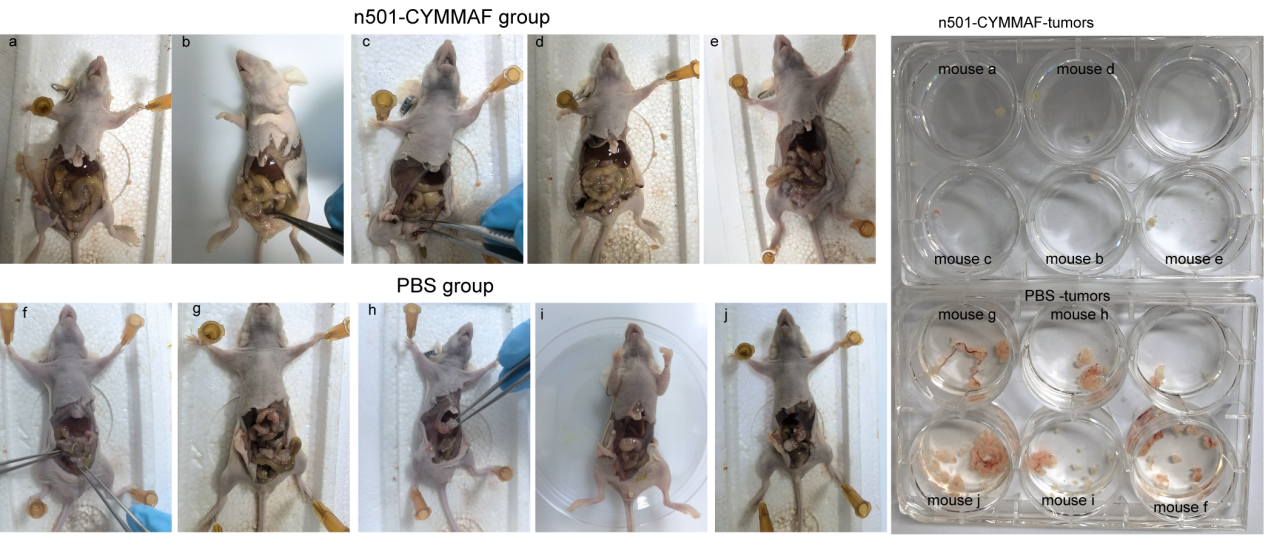


Figure S15. Anatomy of n501-CYMMAF and PBS group and corresponding peeled tumors.


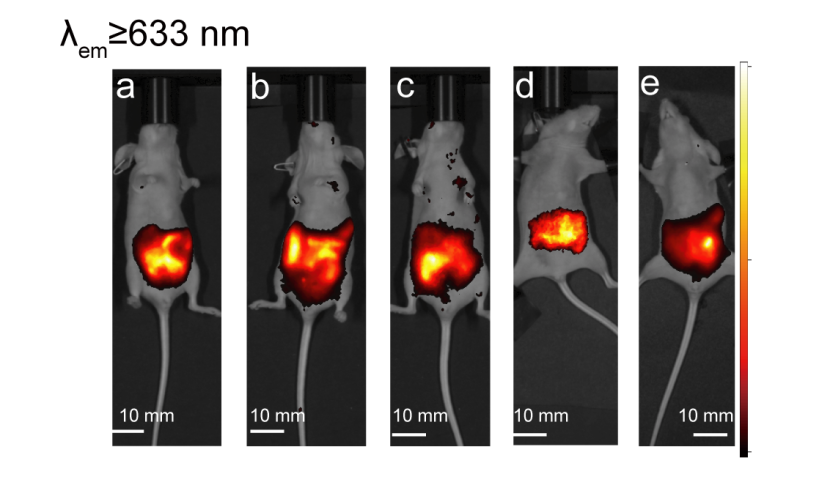


Figure S16. In-vivo fluorescent bioimaging at λ_em_ ≥ 633 nm channel for n501-CYMMAF group at 45^th^ day.


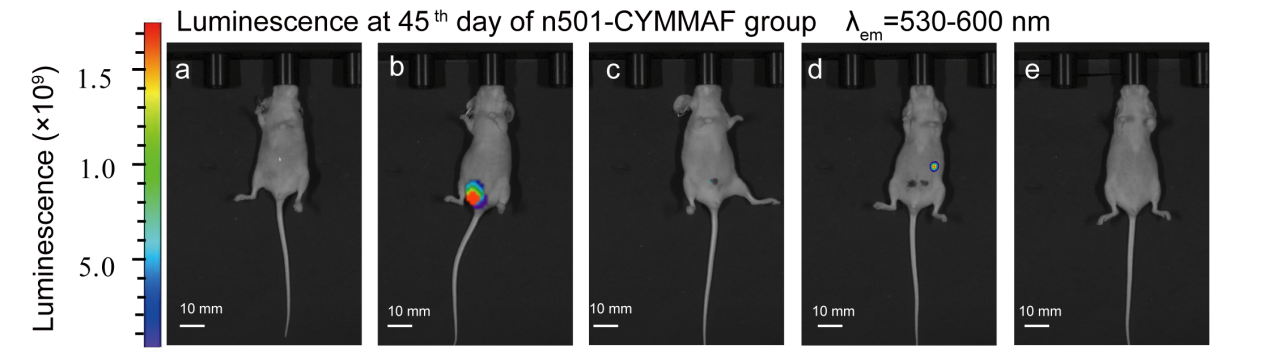


Figure S17. In-vivo luminescent bioimaging based on D-(-)-luciferin for n501-CYMMAF group at 44^th^ day.


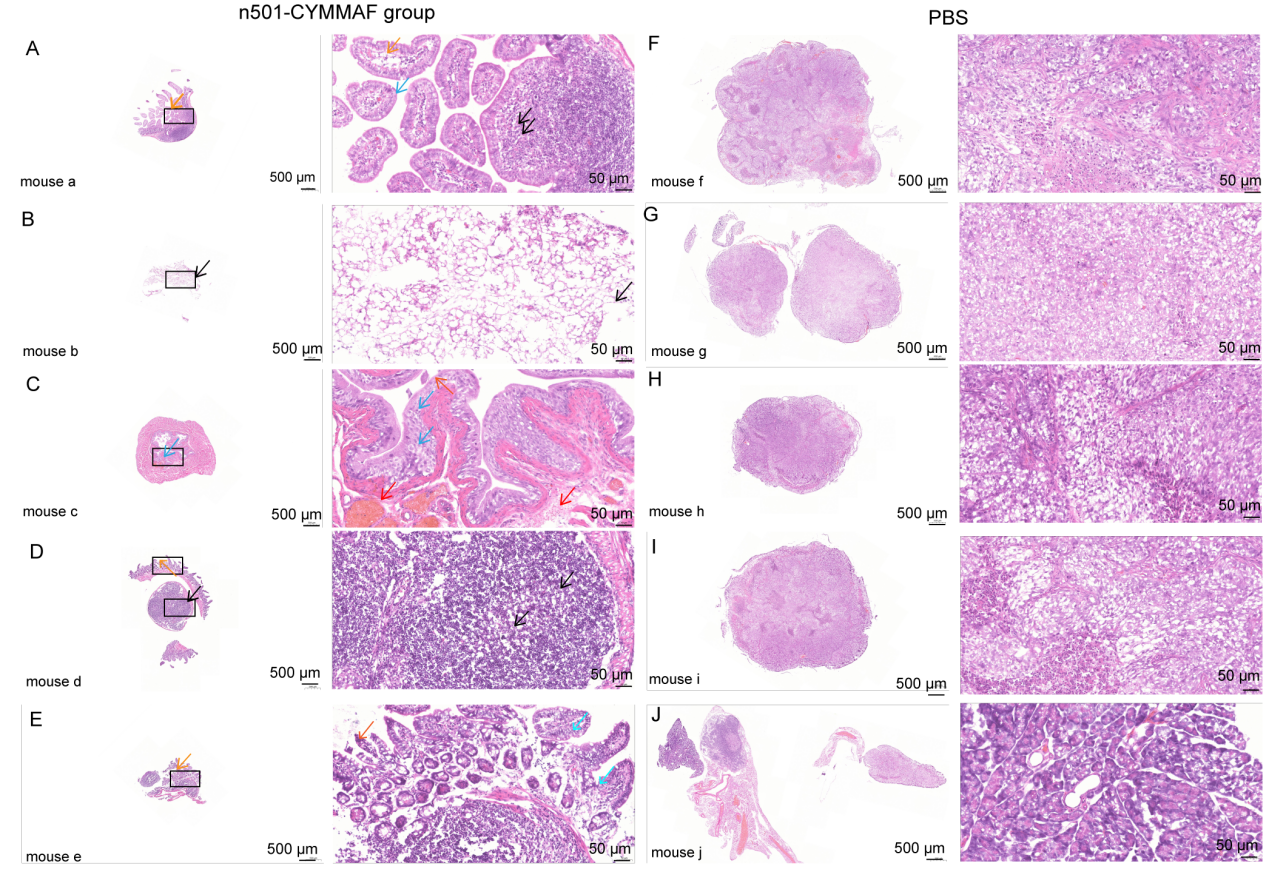


Figure S18. HE analysis of tissues and tumors from n501-CYMMAF and PBS group.

**Reference**

1. McGavin, J.K. & Spencer, C.M. Gemtuzumab ozogamicin. *Drugs* **61**, 1317-1322 (2001).

2. Younes, A., Yasothan, U. & Kirkpatrick, P.J.N.r.D.d. Brentuximab vedotin. *Nat. Rev. Drug Discov.* **11**, 19 (2012).

3. Barok, M., Joensuu, H. & Isola, J.J.B.c.r. Trastuzumab emtansine: mechanisms of action and drug resistance. *Breast Cancer Res.* **16**, 1-12 (2014).

4. Lamb, Y.N.J.D. Inotuzumab ozogamicin: first global approval. *Drugs* **77**, 1603-1610 (2017).

5. Dhillon, S.J.D. Moxetumomab pasudotox: first global approval. *Drugs* **78**, 1763-1767 (2018).

6. Deeks, E.D.J.D. Polatuzumab vedotin: first global approval. *Drugs* **79**, 1467-1475 (2019).

7. Halford, Z., Anderson, M.K. & Clark, M.D.J.A.o.P. Enfortumab vedotin-ejfv: a first-in-class anti-nectin-4 antibody-drug conjugate for the management of urothelial carcinoma. *Ann Pharmacother.* **55**, 772-782 (2021).

8. Keam, S.J.J.D. Trastuzumab deruxtecan: first approval. *Drugs* **80**, 501-508 (2020).

9. Syed, Y.Y.J.D. Sacituzumab govitecan: first approval. *Drugs* **80**, 1019-1025 (2020).

10. Deeks, E.D.J.D. Disitamab vedotin: first approval. *Drugs* **81**, 1929-1935 (2021).

11. Lee, A.J.D. Loncastuximab tesirine: first approval. *Drugs* **81**, 1229-1233 (2021).

12. Markham, A.J.D. Tisotumab vedotin: first approval. *Drugs* **81**, 2141-2147 (2021).

13. Heo, Y.-A.J.D. Mirvetuximab soravtansine: first approval. *Drugs* **83**, 265-273 (2023).

14. Markham, A.J.D. Belantamab mafodotin: first approval. *Drugs* **80**, 1607-1613 (2020).

15. Xu, B. et al. (American Society of Clinical Oncology, 2024).

16. Kharbanda, M., Mishra, H., Asati, V.J.I.J.o.M. & Oncology, I. Efficacy and safety of cetuximab sarotalocan in recurrent/locally advanced head-and-neck cancer: A comprehensive review. *Signal Transduction Targeted Ther.*, 1-4 (2024).

17. Yin, Y. et al. Sacituzumab tirumotecan in previously treated metastatic triple-negative breast cancer: a randomized phase 3 trial. 1-7 (2025).

18 H. Yang, et al., *J. Am. Soc. Mass Spectrom.* 2009, 20, 2284.

19 J. J. Gorman, et al., *Mass. Spectrom. Rev.* 2002, 21, 183.
